# PD-1 combined with TRBC1 and pan-T cell antibodies for robustly monitoring angioimmunoblastic T-cell lymphoma

**DOI:** 10.3389/fmed.2022.962428

**Published:** 2022-09-08

**Authors:** Chunyan Wang, Li Zhu, Songya Liu, Shujuan Yi, Min Xiao, Yicheng Zhang, Xia Mao

**Affiliations:** Department of Hematology, Tongji Medical College, Tongji Hospital, Huazhong University of Science and Technology, Wuhan, China

**Keywords:** multiparameter flow cytometry, angioimmunoblastic T-cell lymphoma, immunophenotyping, PD-1, TRBC1, pan-T antibodies

## Abstract

**Background:**

The diagnosis of AITL is challenging. It may be delayed or even missed due to critical clinical conditions and its histologic and immunophenotypic overlap with other neoplastic and reactive lymphoid proliferations.

**Objective:**

The key objective is to obtain an efficient diagnosis, sensitive disease monitoring and treatment efficacy assessment of AITL using multiparameter flow cytometry (MFC).

**Methods:**

In total, 167 de novo AITL patients were immunophenotypically profiled using sensitive MFC. We precisely identified the aberrant T-cell populations of AITL and performed an in-depth description of their phenotypic characteristics in comparison with their residual normal CD4+ T cells. A comparison of Programmed death receptor-1 (PD-1) expression was performed among AITL and other T-cell lymphomas.

**Results:**

MFC detected a neoplastic T-cell population in 94.1% (80/85) of tissue, 71.5% (108/151) of bone marrow (BM), 100% (8/8) of peripheral blood (PB) and 78.6% (11/14) of body fluid samples. The most frequent immunophenotypic aberrations included the absence and diminished expression of CD3 (71.25% in tissues, 71.3% in BM, 75% in PB, 81.8% in hydrothorax and ascites specimens), followed by the loss or partial loss of CD7 (71.25% in LN, 67.6% in BM, 50% in PB, 81.8% in hydrothorax and ascites specimens). The immunophenotyping of neoplastic T-cell populations showed a high degree of similarity among different sites of the same patient and they might change over time but were relatively stable. Bright PD-1 expression showed high sensitivity and specificity in differentiating AITL from other T-cell lymphomas. In 14 AITL patients, neoplastic T-cell populations were initially missed by T-cell screening tube but were successfully discovered by bright PD-1 expression.

**Conclusion:**

T-cell screening tube can reliably screen neoplastic T-cell populations in AITL patients with typical immunophenotyping, such as loss of surface CD3 and loss of CD7 with a relatively high ratio. Bright PD-1 expression is essential for identifying aberrant T cells in almost all AITLs. The clonality assessment antibody TRBC1 is efficient for robustly and cheaply assessing T-cell clonality. Using PD-1 and TRBC1 combined with pan-T cell antibodies can make a precise diagnosis of AITL and also sensitively monitor minimal residual disease regardless of the antigenic drift of the neoplastic T cells.

## Introduction

Angioimmunoblastic T-cell lymphoma (AITL) is one of the most common types of nodal peripheral T-cell lymphoma (PTCL), accounting for 1–2% of all non-Hodgkin lymphomas (NHLs) and ~15 to 20% of PTCLs (1, 2). In the 4th edition of the WHO classification of lymphoid tumors in the 2017 revision, AITL resides under the umbrella of PTCLs with a follicular helper T-cell (TFH) phenotype, which is defined as positive immunostaining for at least two of the following TFH markers: CD10, Bcl-6, PD-1, CXCL13, and CXCR5, ICOS, and SAP (3). AITL has an aggressive clinical course. Despite advances in treatment approaches, its prognosis remains poor, with 5-year overall survival (OS) and progression-free survival (PFS) estimates of ~32 and ~18%, respectively (4). In addition, the diagnosis of AITL is still challenging due to its invasive course and the overlap between AITL and other neoplastic and reactive benign lesions. Moreover, some patients may present with coagulation disorders and severe cytopenia and are consequently unable to provide tissue samples for pathological diagnosis. As such, an efficient diagnosis requires more auxiliary examinations.

Multiparameter flow cytometry (MFC) is an extremely versatile tool for the diagnosis and monitoring of minimal residual disease (MRD) of hematologic malignancies. Any specimens that can be prepared into a single cell suspension can be detected by MFC, and MFC has unique advantages for body fluid samples. In clinical practice, a single tube with fluorescently labeled antibodies against CD2, surface CD3 (sCD3), CD4, CD5, CD7, CD8, CD45, and CD56 is routinely used to screen phenotype abnormalities of T cells and NK cells (5–7). Regarding AITL, loss and diminished expression of sCD3 on T cells are the most typical phenotypic abnormalities, followed by loss of CD7, and aberrant CD10 expression is distinctive to AITL (7–12). However, CD10 expression has a relatively low sensitivity, and it can also be observed on non-neoplastic T cells that undergo apoptosis and germinal center (GC)-TFH in normal secondary lymphoid organs in follicular lymphoma (FL) (13–15).

TFHs have been identified as the cell of origin of AITL. TFH markers such as programmed death receptor (PD-1), CXCR5, CD10, and ICOS were described as positive in AITL, mostly by immunohistochemistry (2, 16–20). Bright PD-1 expression on aberrant T cells has high sensitivity and specificity in AITL by MFC (21). However, CD4-positive T cells with bright PD-1 expression can also be observed in T-cell/histiocyte-rich large B-cell lymphoma (TCRLBCL) and FL, which may pose challenges for pathological diagnosis. MFC can recognize TFH cells and exclude B-cell lymphoma simply by assessing light chain clonality. Furthermore, CD4-positive T cells with bright PD-1 expression can also be seen in reactive lymphoid hyperplasia (RH) and Hodgkin's lymphoma (HL). Therefore, T-cell clonality assessment is essential to confirm the abnormality of suspicious T cells. Molecular TCR analysis by multiplex polymerase chain reaction (PCR) is routinely used to detect the clonality of T cells (22). In recent years, the Beta Mark TCR Vbeta Repertoire Kit and T-cell Receptor Constant β Chain-1 (TRBC1) by flow cytometry have provided an opportunity to facilitate the detection of clonal TCR αβ T cells (5, 23–25).

At the time of initial diagnosis, when BM, PB and body fluid specimens are the first or the only specimen obtained for diagnosis, whether pan-T cell antibodies in a single tube are adequate to screen aberrant T cells for AITL and the potential diagnostic pitfalls have not been well-elaborated. We aimed to improve the early diagnosis of AITL and reduce the rate of misdiagnosis and missed diagnosis. A total of 167 de novo AITL patients were included for reanalysis. Other T-cell lymphomas and some HL, FL and RH in the same period were set as control cases. When the T- and B-lymphocyte screen tubes were negative, an additional tube with a combination of TFH markers (PD-1 and CD10) and some pan-T cell antibodies (such as CD3, CD4, CD5, CD7, and CD8) was added to confirm or exclude a supposed PTCL diagnosis, and for patients with an equivocal clinical history or immunophenotyping, TRBC1 was added to this tube to determine the clonality of the T cells.

## Materials and methods

### Patients, controls, and samples

A total of 258 samples obtained from 167 de novo AITL patients (120 men and 47 women; median age 59 years, range 19–82) from 2014 to 2021 were included from the database of the Department of Hematology of Tongji Hospital, Tongji Medical College, Huazhong University of Science and Technology. Other patients, including 10 with anaplastic large cell lymphoma (ALCL), 20 with other PTCLs, 6 with mycosis fungoides (MF), 1 with Sezary syndrome, 1 with adult T-cell leukemia/lymphoma (ATLL), 3 with lymphocyte-variant hypereosinophilic syndrome (L-HES) and 3 with T-cell clones of uncertain clinical significance (T-CUS), were immunophenotypically profiled as controls. Twenty-nine tissue samples with reactive lymphoid hyperplasia (RH), three with nodular lymphocyte-predominant Hodgkin lymphoma (NLPHL), three with follicular lymphoma (FL) and 3 with T-cell/histiocyte-rich large B-cell lymphoma (TCRLBCL) were also detected by MFC. The AITL specimens included 151 BM, 85 tissue, 14 body fluid and 8 PB samples. Ninety-seven follow-up samples obtained from 37 AITL cases were reanalyzed. The maximum time interval between the follow-up samples and the initial ones was 6.5 years. PD-1 was added to 69 patients with AITL and the above control patients. TRBC1 was added to 38 patients with AITL, 6 with ALCL, 2 with HES, 13 with PTCL, 3 with MF, and 3 with T-CUS. The specimens included bone marrow (BM), peripheral blood (PB), tissue samples (lymph node, fine needle aspiration, tonsil, spleen, skin, etc.) and body fluid specimens (hydrothorax and ascites). Basic information of these patients and samples was listed in Table 1. All cases were reviewed and confirmed by two of the authors (X.M. and C.Y.W). All tissue specimens had pathological and immunohistochemical analysis of paraffin sections, and most of them had genotypic studies. The diagnosis was made in accordance with the 2017 World Health Organization. Appropriate informed consent was obtained from all patients prior to specimen collection in accordance with the Declaration of Helsinki, and the research protocol was approved by the ethics committees of Tongji Hospital.

**Table 1:** Clinical information of patients.

	**AITL** **(*n* = 167)**	**ALCL** **(*n* = 10)**	**Other PTCLs** **(*n* = 20)**	**MF** **(*n* = 6)**	**Sesary** **(*n* = 1)**	**ATLL** **(*n* = 1)**	**HES** **(*n* = 3)**	**T-CUS** **(*n* = 3)**
**Sample**	258	12	22	6	1	1	3	3
BM	151	4	20	4	1	1	3	3
PB	8	0	0	0	0	0	0	0
Tissue	85	8	2	2	0	0	0	0
Body fluid	14	0	0	0	0	0	0	0
**Sex**								
Male	120	8	16	5	0	0	1	1
Female	47	2	4	1	1	1	2	2
**Age**								
Age range	19–82	7–70	32–83	51–63	-	-	4–56	58–83
Median age	59	33.5	54	57.5	-	-	39	67

BM, bone marrow; PB, peripheral blood; AITL, angioimmunoblastic T-cell lymphoma; ALCL, anaplastic large cell lymphoma; PTCL, peripheral T-cell lymphomas; MF, mycosis fungoides; ATLL, adult T-cell leukemia/lymphoma; L-HES, lymphocyte-variant hypereosinophilic syndrome; T-CUS, T-cell clones of uncertain clinical significance; “-,” absent.

### Flow cytometry

#### Panels

All of the samples were stained with the T- and B-cell lymphoid screening tube (LST) described below. A cocktail of anti-human monoclonal antibodies, including CD45-V500, CD2-FITC, CD3-APC-Cy7, CD4-V450, CD5-PE-Cy7, CD7-PE, CD8-APC, and CD56-ECD, was used to screen mature T- and natural killer (NK)-cell lymphoproliferative disorders. Monoclonal antibody combinations containing CD45-V500, CD19-V450, CD20-APC, CD38-PerCP-Cy5.5, CD10-PE-Cy7, kappa-FITC, and lambda-PE were used to screen mature B-cell lymphoproliferative disorders for patients under 30 years old, and CD45-V500, CD19-V450, CD20-APC-Cy7, CD38-PercP, CD56-ECD, CD138-APC, cytoplasm kappa-FITC, and cytoplasm lambda-PE were combined in one tube to serve as B- and plasma-cell screening tubes for patients more than 30 years old. PD-1 (EH12.2H7, BioLegend, APC), CD10, TRBC1 (JOVI.1, Caprico biotechnologies, FITC) and some pan-T cell antibodies (such as CD3, CD4, CD5, CD7, and CD8) were placed in an additional tube to confirm or exclude a supposed PTCL diagnosis and for patients with an equivocal clinical history or immunophenotyping. If CD4+ T cells had aberrant immunophenotyping detected by T-cell screening tube, then fluorescent labeled antibodies CD103, CD25, CD57, CD10, CD30, CD99, CD1a, CD45RA, CD45RO, cCD3, ki67, CD26, PD-1, TCR aβ, TCRγδ, and TRBC1 were subsequently added.

#### Specimen procedures

EDTA-anticoagulated BM, PB samples and body fluid samples were freshly (<4 h) processed. A total of 2 × 10^6^ cells per tube were added and incubated with monoclonal antibodies for 15 min at room temperature. Erythrocytes were lysed with 1 × Lysing Solution (BD Biosciences, San Jose, CA) at room temperature for 15 min using a standard lyse/wash technique. Tissue specimens were stored in RPMI media after isolation and transported to our laboratory within 2 h. A single-cell suspension was prepared immediately after its arrival. For FNA or LN with a diameter <2 mm, they were minced into small fragments using scissors and gently ground on metal gauze. The relatively larger LNs were prepared by a gentleMACS Dissociator (Miltenyi Biotec). Then, the single-cell suspension was filtered through a 38-μm filter cloth and centrifuged at 1,300 rpm for 5 min. The supernatant was discarded, and the cells were resuspended again using 2 mL of RPMI. A total of 2 × 10^6^ cells (or less when the cell count was extremely low) per tube were stained with monoclonal antibodies for 15 min at room temperature. Erythrocytes were lysed with 1 × Lysing Solution (BD Biosciences, San Jose, CA) at room temperature for 6 min. Kappa and lambda tubes need to be incubated in a 37°C water bath first and then washed three times with PBS before staining with antibodies. Cytoplasmic antibodies were applied after the surface antibodies were fixed, and the membrane was disrupted with a BD IntraSure Reagent Kit (BD Biosciences, San Jose, CA).

#### Immunophenotyping

T cell populations with the loss and/or decreased expression of one or more pan-T cell markers or expression those at abnormal density were considered as aberrant populations (26, 27). In routine practice, T cells were considered aberrant consisting of the following: (1) abnormal expression (loss, enhanced, or dimished) of pan-T cell markers such as surface CD5, CD2, CD3, or CD7 antigens and cytoplasmic CD3 (cyCD3); (2) CD4/CD8 ratio >10 or <0.1 and had a predominant CD4+/CD8+ or CD4–/CD8– cell population; (3) expression of non-T cell markers including CD10, CD30, CD56, TdT, CD34, and TCR γδ (28). B cell subpopulations were defined as aberrant by the presence of 2 main types of phenotypic abnormality: immunoglobulin light chain class restriction (κ/λ ratios >3:1 or <0.3:1, or more than 25% of mature B cells lacking or expressing low level surface immunoglobulin) and/or antigenic aberrancies (29). TRBC 1 is regarded as monoclonal expression when the positive rate is higher than 85 or <15%. Normal subsets of T cells should be recognized and excluded, including some normal γδ T cells with positive CD3 expression and negative CD5, CD4, and CD8 expression (26). The absence of CD3 and CD5 expression of CD4+ mature T cells always suggested aberrant, loss of CD7 require distinguish from normal CD4+CD7- T cells. The pan-T cell antibodies and PD-1 mean fluorescence intensity (MFI) of the aberrant T-cell populations and residual CD4+ T cells were evaluated. The immunophenotyping and ratio of neoplastic T cells were evaluated in different samples from the same patient, and the phenotypic changes were followed after therapy. We used BD FACS LSRFortessa and a Canto II cytometer (BD Biosciences, San Jose, CA) for data collection. BD diva software was used for data analysis.

#### Statistical analysis of the immunophenotypic data

The Mann–Whitney U test was applied for the statistical comparison between different groups. Statistical analyses were carried out using GraphPad Prism Viewer 8.0.1 software applying a significance level of *p* < 0.05.

## Results

### Sensitive multiparameter flow cytometry improves the identification of AITL

In our report, MFC detected a neoplastic T-cell population of AITL in 94.1% (80/85) of tissue, 71.5% (108/151) of bone marrow (BM), 100% (8/8) of peripheral blood (PB) and 78.6% (11/14) of body fluid samples. The ratio of aberrant T-cell populations in total nucleated cells was significantly higher (*P* < 0.05) in tissue samples (0.4–61.5%, median 9.15%) than in BM (0.016–17.2%, median 0.38%) and PB specimens (0.03–5.9%, median 0.85%). Four patients were clinically diagnosed with T- and B-cell composite lymphoma due to inconsistencies between the pathology and flow cytometry, with AITL by histopathology and B-cell lymphoma by MFC (three DLBCL and one SLL). Two patients had T/B-cell composite lymphoma detected by MFC and histopathology. Three patients were previously diagnosed with DLBCL and were ultimately diagnosed with AITL by histopathology, MFC and molecular analysis. Three patients were previously diagnosed with HL and were ultimately diagnosed with AITL by histopathology and MFC.

### T-cell screening tube can reliably screen neoplastic T-cell populations in AITL patients with typical immunophenotyping, such as a loss of surface CD3 and a loss of CD7 at a relatively high ratio

**Figure 1:**
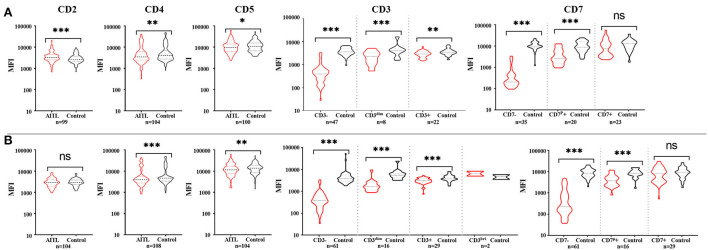
The expression features of pan-T cell antigens of AITL patients in tissue **(A)** and bone marrow **(B)**. **(A)** Compared with the corresponding non-neoplastic CD4+ T cells in AITL, neoplastic T cells had bright CD2 expression and diminished CD3, CD4, and CD5 expression. CD7 mean fluorescent intensity (MFI) had no significant difference between CD7+ neoplastic T cells and normal CD4+ T cells. **(B)** In bone marrow samples, neoplastic T cells had obviously diminished CD3, CD4, and CD5 expression, and there were no significant difference of CD2 expression between neoplastic T cells and normal CD4+ T cells. CD7 mean fluorescent intensity (MFI) had no significant difference between CD7-positive neoplastic T cells and normal CD4+ T cells. *p < 0.05, **p < 0.01, ***p < 0.001.

**Figure 2:**
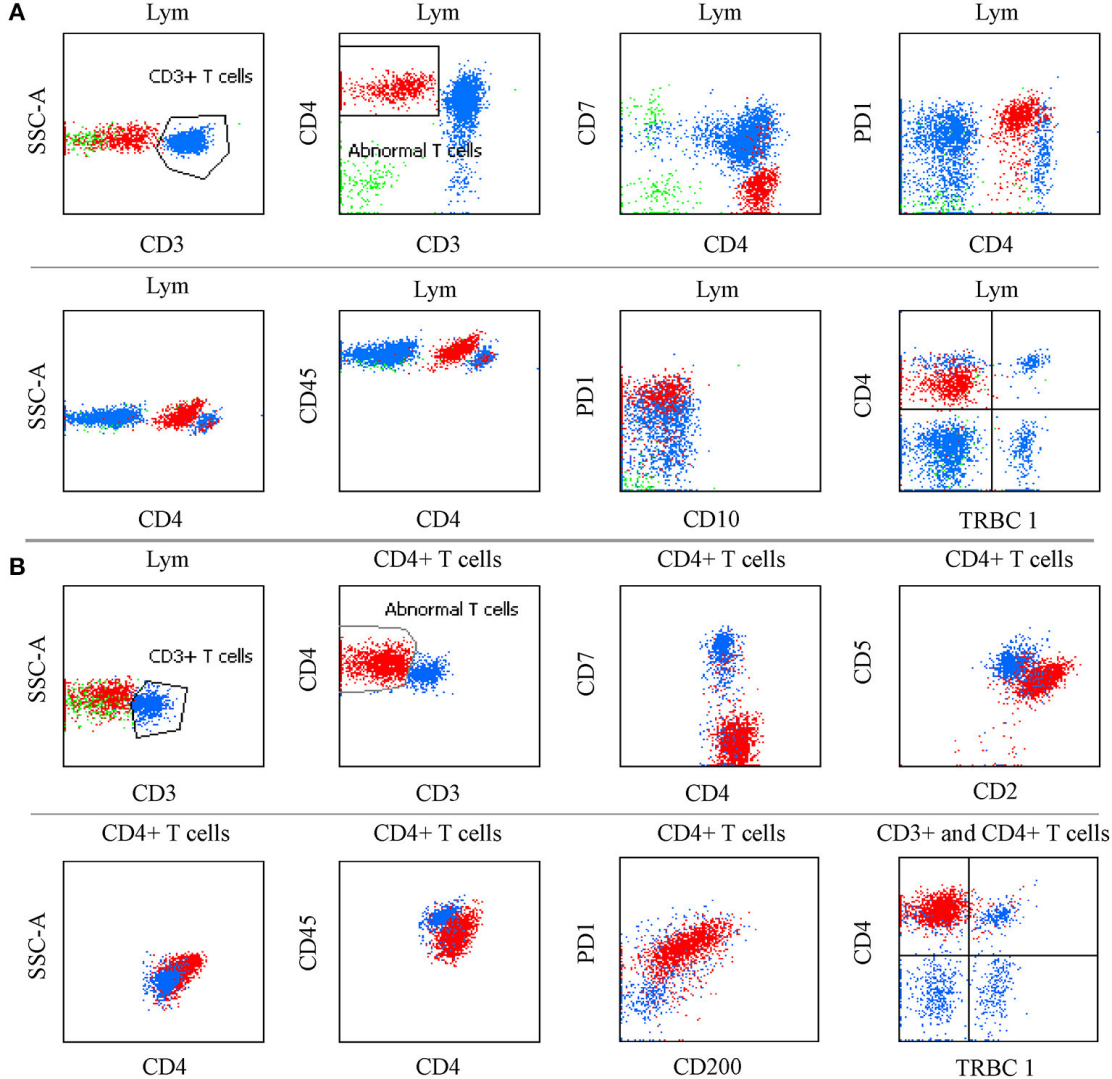
The most common aberrancies of pan-T cell antigens in AITL. **(A)** PB specimen in which neoplastic CD4+ T cells had bright CD45 and PD-1 expression, and diminished CD4 expression, lack of CD3, CD7, and TRBC1 with slightly larger side scatter. The same immunophenotyping was observed in BM, PB, and LN specimens. **(B)** FNA sample in which CD4+ aberrant T cells had bright CD4, CD2, and PD-1 expression, CD200 was positive, CD5, and CD45 were diminished; CD3, CD7, and TRBC1 were absent; Side scatter was enlarged.

We considered only one specimen per site per patient and analyzed the above antigens. In the T-cell screening tubes, the most frequent immunophenotypic aberrations were the absence and diminished expression of CD3 (71.25% in tissues, 71.3% in BM, 75% in PB, 81.8% in hydrothorax and ascites specimens), followed by the loss or partial loss of CD7 (71.25% in LN, 67.6% in BM, 50% in PB, 81.8% in hydrothorax and ascites specimens). Bright CD3 expression was seen in two patients. The expression of CD3 and CD7 in different specimens is listed in Table 2. The coexistence of surface CD3-positive and -negative neoplastic populations was noted in 4.5% (8/167) of patients. The expression of CD45 and SSC was heterogeneous. Bright CD45 expression and large SSC were commonly observed. CD45 negativity was seen in one patient. The CD5 MFI of CD4+ T-cell populations was higher than that of CD8+ T cells by visual assessment. In AITL, CD4 and CD5 MFI were lower in aberrant T-cell populations than in normal CD4+ T cells and were more obvious in BM samples than in tissue specimens (Figure 1). CD2- and CD5-negative cells were observed in 0.6% (1/167) of patients. In BM samples, there was no significant difference in CD2 MFI between neoplastic and normal CD4+ T cells, whereas in tissue specimens, aberrant T-cell populations had brighter CD2 expression than normal controls. CD4-CD8- tumor T cells were detected in 1.8% (3/167) of patients. CD4-CD8+ immunophenotyping was found in 0.6% (1/167) of patients. CD56 was positive in 3.0% (5/167) of patients and was partially expressed in 1.8% (3/167) of patients. We discovered that lack of one expected pan-T cell marker is not enough, at least two (preferably three) of the following indicator of CD4+ T cells must be aberrant: the location of CD45/SSC, the MFI changes (mainly dimished or loss) of CD3, CD5, CD7, CD4 and CD2. The most common aberrancies of pan-T cell antigens in AITL were shown in Figure 2.

**Table 2:** The expression of CD3 and CD7 in different specimens.

	**Tissue 94.1% (80/85)**	**BM 71.5% (108/151)**	**PB 100%(8/8)**	**Pleuroperitoneal fluid 78.6% (11/14)**
**CD3**				
Bright	0	1.85% (2/108)	0	0
Positive	28.75%(23/80)	26.85%(29/108)	25.0% (2/8)	18.2%(2/11)
Dim or partial	11.25% (9/80)	14.8%(16/108)	37.5% (3/8)	0%(0/11)
Loss	60.0% (48/80)	56.5%(61/108)	37.5% (3/8)	81.8%(9/11)
**CD7**				
Positive	28.75% (23/80)	32.4% (35/108)	50% (4/8)	18.2% (2/11)
Partial	26.25% (21/80)	24.1% (26/108)	25.0% (2/8)	27.3% (3/11)
Loss	45.0% (36/80)	43.5% (47/108)	25.0% (2/8)	54.5% (6/11)

### Bright PD-1 expression showed high sensitivity and specificity in differentiating AITL from non-AITL PTCLs and HES and was better identified than CD3 and CD10

**Figure 3:**
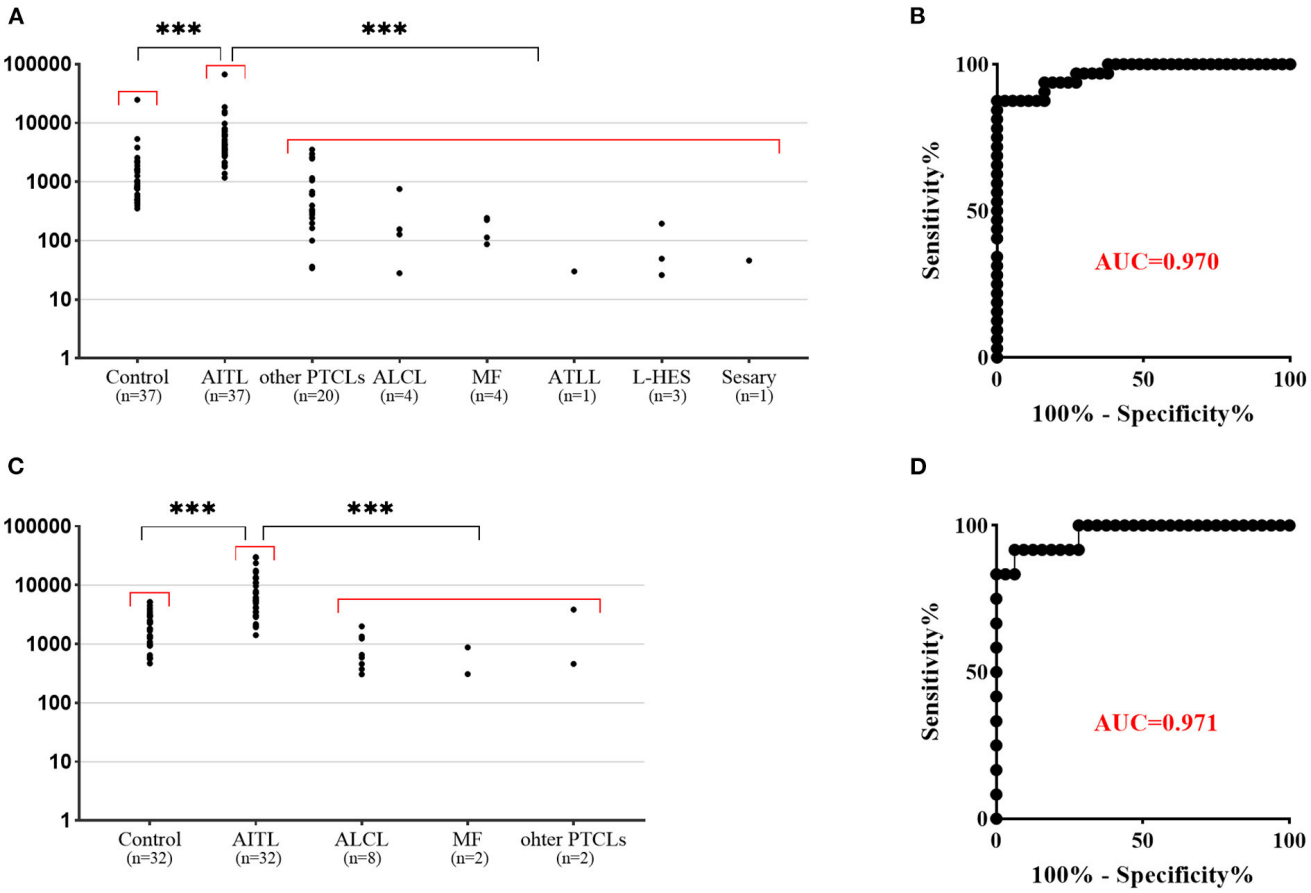
Flow cytometric findings of PD-1 expression in non-neoplastic CD4+ T cells and T-cell lymphoma. **(A)** PD-1 mean fluorescent intensity (MFI) in bone marrow samples. The corresponding non-neoplastic CD4+ T cells in AITL were used as a control. The PD-1 MFI in AITL is significantly higher than that in control T cells and other T-cell lymphomas. **(B)** ROC curve of PD-1 MFI bone marrow samples shows high sensitivity and specificity in differentiating AITL from non-AITL T-cell lymphoma (*p* < 0.001, AUC 0.970: sensitivity 100.0% and specificity 83.33% for a cutoff of MFI 1162). **(C)** PD-1 mean fluorescent intensity (MFI) in tissue samples. The PD-1 MFI in AITL is significantly higher than that in control T cells and other T-cell lymphomas. **(D)** ROC curve of PD-1 MFI tissue samples shows high sensitivity and specificity in differentiating AITL from non-AITL T-cell lymphoma (*p* < 0.001, AUC 0.971: sensitivity 91.67% and specificity 93.75% for a cutoff of MFI 2023). ***p < 0.001.

The PD-1 mean fluorescence intensity of aberrant T cells was significantly higher than that of residual normal CD4+ T cells (*P* < 0.0001). In ALCL, PTCLs, MF, ATLL, L-HES and T-CUS, PD-1 was negative to medium intensity expression and rarely had bright PD-1 expression. The PD-1 mean fluorescent intensity (MFI) in different subgroups is shown in Figure 3. The ROC curve (Figure 3) of PD-1 MFI in tissue and BM samples also showed high sensitivity and specificity in differentiating AITL from non-AITL T-cell lymphomas and HES (sensitivity 91.67% and specificity 93.75% for a cutoff of MFI 2023 in tissue samples, sensitivity 100% and specificity 83.33% for a cutoff of MFI 1162 in bone marrow specimens). Although the cutoff values were different between tissue and bone marrow samples, there was no significant difference in PD-1 MFI between them (*P* > 0.05). Aberrant T-cell populations were better identified by distinctive bright PD-1 expression than by CD3 and CD10 for AITL screening. In our study, CD10 expression (partial or positive) was only observed in 43.8% (35/80) of BM and 45.1% (32/71) of tissue specimens, which was much lower than that in a previous study. CD10 expression detected by MFC in tissue samples was relatively different from that detected by histopathology.

**Figure 4:**
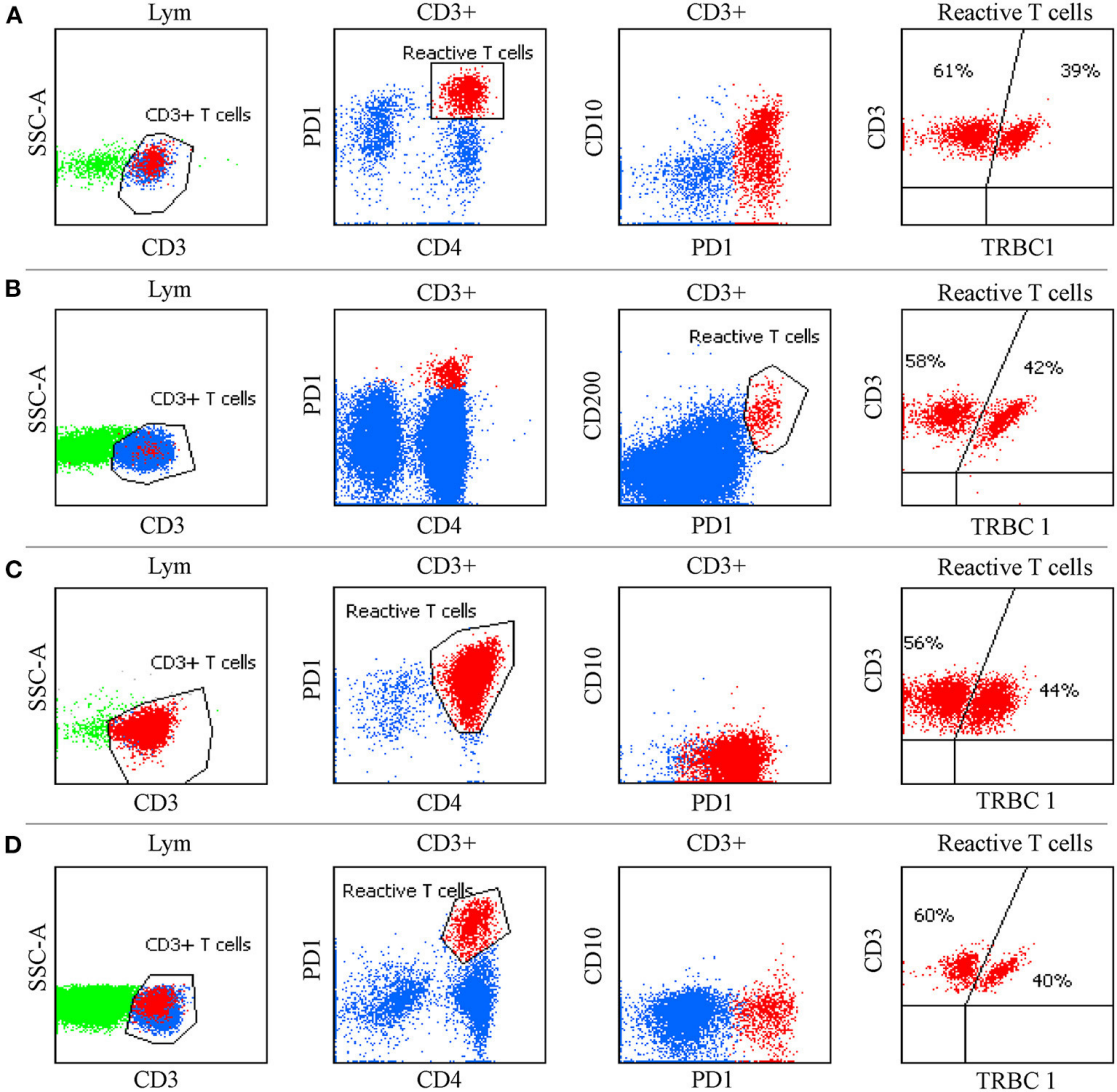
Flow cytometric findings in non-T cell lymphoma. **(A)** CD4+ T cells with CD10 and bright PD-1 expression were observed in follicular lymphoma. **(B)** CD4+ T cells with CD200 and bright PD-1 expression were seen in tuberculous granuloma. **(C)** Expansion of CD4+T cells with bright PD-1 expression was present in nodular lymphocyte-predominant Hodgkin lymphoma. **(D)** CD4+ T cells with distinctive bright PD-1 expression were observed in reactive follicular hyperplasia. TRBC1 was polyclonal in these populations.

It is worth noting that CD4+ T-cell subgroups with distinctive bright PD-1 expression could be commonly observed in RH, HL and some FL tissue samples, but were less frequently seen in BM (Figure 4). CD4+ T-cell populations with CD10 and bright PD-1 expression (an immunophenotype mimicking AITL) were also observed in 2 LN specimens (one with FL and one with RH) but were demonstrated to be polyclonal by TRBC1. The FL patient had an aggressive clinical course with fever, a large amount of polyclonal hyperimmunoglubin, EBV infection, polyserositis and retroperitoneal LN enlargement, which was highly suggestive of AITL. Retroperitoneal FNA histopathology suggested that high-grade FL and TCR/IgH gene rearrangement of FNA were negative, and few cells exhibited Bcl-6 gene rupture and rearrangement by fluorescence *in situ* hybridization. The MFC was negative in both the T-cell and B-cell screening tubes. Another patient had a history of recurrent lymphadenopathy, and the LN histopathology showed RH. MFC, karyotype analysis and next-generation sequencing were negative. In the above cases, clonality assessment was essential to exclude T-cell lymphoma.

### TRBC1 was polyclonal in reactive conditions and was monoclonal in αβ+PTCLs

None of the AITL patients expressed TCR γδ, and as mentioned in our previous paper, nearly 57% of patients lost the expression of TCR αβ (30). We performed TCR Vbeta in one case expressing TCR αβ and confirmed the monoclonality of the abnormal T cells. TRBC1 was added to 38 AITL patients, 6 with ALCL, 2 with HES, 13 with PTCL, 3 with MF, and 3 with T-CUS. Only 14.3% (5/38) of AITL patients had diminished expression of TRBC1, and meantime neoplastic T cells were all express CD3 and TCR αβ. In 57 TCR αβ negative cases, 5 cases were CD3 positive with TRBC1 negative (4/4), and the remaining 52 cases were CD3 negative and among them TRBC1 was all absent (14/14). In 42 TCR αβ positive cases, 30 cases were CD3 positive and among them TRBC1 was 50% positive (5/10), and the rest 12 cases were CD3 negative (TRBC1 was not added). Among 10 cases of other T-cell lymphomas who had detected all of these three markers, TRBC1 was seen positive in 1 case with CD3 and TCRαβ negative and three cases with CD3 and TCRαβ positive. TRBC1 was negative in 4 cases with CD3 and TCR αβ positive and two patients with CD3 and TCR αβ negative. The aberrant T-cell populations in TCR αβ+ T-cell lymphoma were monoclonal. Polyclonal expression of TRBC1 has never been observed on aberrant T cells in AITL and other PTCLs. Normal CD4+ T lymphocytes corresponding to AITL tumor cells and the normal CD4+CD7- T-cell population in BM, PB and tissues were polyclonal. TRBC1 can robustly exclude reactive polyclonal T cells. In addition, sCD3-CD4+ T-CUS was observed in a 58/male chronic myelogenous leukemia patient, a 67/male severe pneumonia patient and an 83/female iron deficiency anemia patient, and TRBC1 was negative for aberrant T cells. In 3 HES patients, TCRαβ and TRBC 1 was both negative (the flow of two L-HES cases was provided in Supplementary material).

### Some AITLs were initially missed by T-cell screening tubes, but neoplastic T-cell populations were successfully discovered by bright PD-1 expression

**Figure 5:**
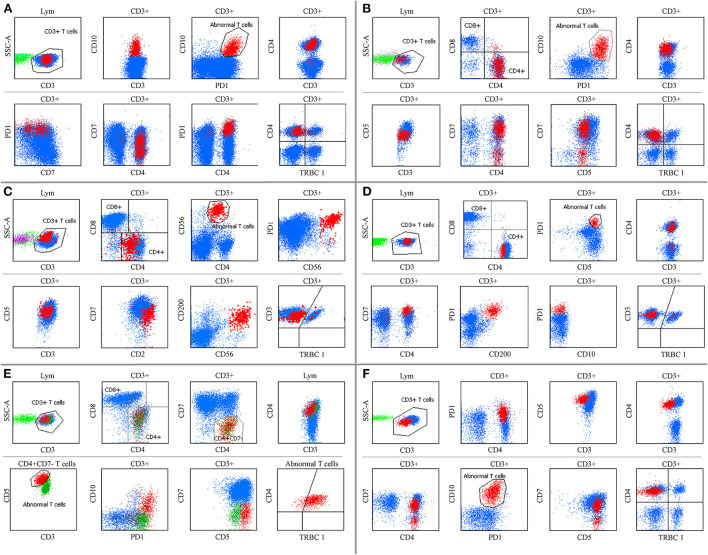
Some AITL immunophenotyping could not be detected by the T-cell screen tube. **(A,D,E,F)** show BM specimens. **(B,C)** show tissue samples. **(A)** Aberrant T cells were CD3+, CD4+, and CD10+ with bright PD-1 expression, and CD7 was partially expressed. **(B)** Neoplastic T cells were CD4+, CD7+, and CD10+ with slightly diminished CD3 and CD5 expression and bright PD-1 expression. **(C)** Tumor cells were CD3+, CD5+, CD7+, and CD200+ with diminished CD4 expression and bright CD2, CD56, and PD-1 expression, and CD10 was negative (not shown). **(D)** Abnormal T cells were CD3+, CD4+, CD5+, and CD200+ with bright CD7 and PD-1 expression, and CD10 was negative. **(E)** An extremely low ratio of CD7 negative T cells was CD3+, CD4+, and CD10+ with bright CD5 and PD-1 expression. The immunophenotyping of neoplastic T cells shifted over time **(E,F)** before and after therapy, respectively. TRBC1 did not show polyclonal expression in these populations.

To patients who were clinically suspected of lymphoma or had equivocal immunophenotyping but had no demonstrable diagnosis at the time of receiving the specimens, PD-1, TRBC1 and some T-cell markers (such as CD3, CD5, CD7, CD4, and CD8) were added to one tube for screening AITL when the MFC did not detect obvious phenotypic abnormalities using T- and B-cell lineage screening tubes. Among them, the T-cell screening tubes initially failed to identify aberrant T cells in 18 samples (seven tissue samples, nine BM, and two PB specimens) from 14 AITL patients, but neoplastic T-cell populations were successfully discovered by bright PD-1 expression. These patients were eventually diagnosed with AITL by histopathology and molecular analysis. The pan-T cell antigens did not show aberrant expression by visual assessment of the T-cell screening tubes. Some of the immunophenotyping characterizations of these patients are summarized in Figure 5. Next generation sequencing (NGS) was performed on 6 of these 14 challenging patients, and all cases had mutations in *TET2*, five patients had mutations in *RHOA (G17 V)*, two patients had mutations in *IDH2*, and *DNMT3A* mutations were seen in one patient.

### Phenotypic changes may appear over time but are relatively stable

We explored immunophenotypic changes over time. Sequential sampling was performed of 31 AITL patients, including four tissue, 75 BM, three ascites, five pleural effusion and seven PB samples. The detection interval ranged from 1 to 78 months after the initial sample. Compared with the initial phenotype, five patients had heterogeneous expression of CD7. Two patients had diminished expression of CD3. One patient had phenotypic changes in both CD3 and CD7 in the second MRD (Figures 5E,F) and had three aberrant T-cell populations with various expression levels of PD-1, CD3, CD10, CD4, and CD7 in the third MRD. Another patient was CD5 negative after CD5 chimeric antigen receptor T-cell therapy. PD-1 was negative in patients with anti-PD-1 therapy. In patients who had two subgroups of neoplastic T cells, the clonal expansion of two phenotypically distinct T-cell populations was variably balanced throughout the course of the disease. Although the initial immunophenotyping of neoplastic T cells could shift over time, at least one major phenotypic abnormality used for gating neoplastic T cells remained unaltered.

### The immunophenotypic features of AITL in different sites

To compare the patterns of pan-T cell antigens expression in different sites, we reanalyzed 66 AITL patients with samples from at least two of the following sites: tissue, bone marrow, peripheral blood, ascites, pleural effusion or cerebrospinal fluid involvement. The pan-T cell antigens expression of aberrant T cells at different sites had a high degree of similarity among the different sites of the same patient, with only slight changes in intensity. The absence of CD3 and CD7 was more often seen in patients with ascites and pleural effusion involvement. Inconsistent expression of CD7 in different types of samples was seen in 9.3% (5/54) of patients and was the most common distinction among samples from different sites. CD3 expression was inconsistent between LN and BM in 11.1% (6/54) of patients without an obvious pattern, although all 6 patients had diminished or negative CD3 expression. In patients expressing CD2, the CD2 MFI of aberrant T cells was higher than that of residual normal CD4+ T cells (*P* < 0.05) in tissue samples, but no significant difference was observed for BM.

## Discussion

There is considerable heterogeneity in the clinical course of AITL, and the time from onset to final diagnosis was between 20 days and 4 years, with survival ranging from 20 days to 10 years. Due to the histopathology overlap between AITL and other neoplastic and reactive benign lesions, patients underwent 1 to 4 tissue biopsies on average before a final diagnosis. The most confusing differential diagnoses were reactive lymphoid hyperplasia, T-cell/histiocyte-rich large B-cell lymphoma and nodular lymphocyte-predominant Hodgkin lymphoma. Sensitive MFC detected a neoplastic T-cell population in 94.1% of tissue samples and 71.5%-100% of BM, PB and body fluid samples, which can improve the identification of AITL. As the immunophenotyping of neoplastic T cells may change over time but are relatively stable, therefore MFC is effective for the evaluation of minimal residual disease following therapy.

When there are no demonstrable T-cell neoplasms, the challenges of establishing the diagnosis of AITL by using pan-T cell antibodies mainly come from the following aspects. Firstly, it is quite clear that in contrast to the abundant background components, such as normal T cells, B cells and plasma cells, the proportion of neoplastic T cells is significantly lower, especially in BM and PB specimens. Secondly, AITL with monoclonal B cells and plasma cells or a large amount of polyclonal plasma cells has been reported before. In our preliminary experience, in 6 cases of composite AITL and B-cell lymphoma, the immunophenotyping of neoplastic T cells rarely lost the expression of sCD3 (1/6). It is easy to emphasize monoclonal B cells and plasma cells and miss the subtle phenotypic abnormalities of the relatively few T cells. Thirdly, the coexistence of two distinct abnormal T-cell populations with and without surface CD3 expression was previously reported and was also observed in our report, which may underestimate the tumor burden if only focusing on the sCD3 loss (21). Fourthly, BM involvement was reported in 28–60% of patients (31). However, the ratio has been underestimated. Normal CD7-negative T-cell subgroups are commonly seen in skin, PB and BM specimens and they may expand in benign dermatomes and other reactive conditions (32, 33). In AITL patients, the CD4-positive and CD7-negative phenotype of T-cell populations were exhibited by a variable percentage of neoplastic cells and the admixture of normal CD4-positive T cells. It is challenging to discover malignant CD7-negative T cells when their proportion is extremely low without aberrant expression of other pan-T cell antigens. Finally, we discovered that some AITL cases only showed bright PD-1 expression with or without CD10 expression (Figure 5) and had extremely subtle or no phenotypic changes in pan-T cell antigens. It is particularly difficult to monitor AITL before a final diagnosis.

In previous studies, neoplastic T-cell populations were identified by aberrant antigen expression, such as the presence or absence of antigens that differ significantly from normal T cells, and increased or decreased intensity of antigen expression in comparison to their normal cell counterparts (26). Sometimes the changes of these indicators are so subtle by visual that they need to be assessed by gating neoplastic T cell subgroups using specific markers. Since bright PD-1 expression shows high sensitivity and specificity in differentiating AITL, an additional tube containing PD-1 was performed on suspected patients even when the T-cell screen tube did not discover abnormalities. Interestingly, we discovered that some AITLs were easily missed by the T-cell screening tubes because of the low ratio of neoplastic T cells and the extremely subtle phenotypic changes in pan-T cell antigens. Moreover, a few of them did not have aberrant expression of pan-T cell antigens by visual assessment (Figures 5A,B,D). These neoplastic T cells can only be identified by bright PD-1 expression. To our knowledge, this is a novel finding in AITL that has never been reported before.

The most common immunophenotypic aberrations of AITL were the loss and diminished expression of sCD3 (71.25–81.8% in different specimens), followed by the loss or partial loss of CD7 (50–81.8% in various samples). However, patients with L-HES may present with a persistent circulating sCD3-CD4+ aberrant T-cell phenotype, and clinical features such as skin involvement (most common), hypereosinophilia, peripheral adenopathy and splenomegaly mimic AITL. Clonal rearrangement of T-cell receptor genes can be observed in half of these patients (34). Moreover, sCD3-CD4+ T-CUS was also observed in other hematological disease except lymphoma. PD-1 was negative in the L-HES and T-CUS patients mentioned above, which was useful to distinguish AITL from L-HES and T-CUS of sCD3-/CD4+ immunophenotyping.

Aberrant T cells with CD10 expression were regarded as unique to AITL in previous studies, and CD10 expression (partial or positive) was reported to be 54% in BM/PB specimens and 88.9% in LN samples (12). The relatively specific marker CD10 had low sensitivity in our study, and the expression of CD10 by flow cytometry was significantly different from that in pathology. The abundant background cells and the confusing follicle germinal center cells in the tissue samples may be potential reasons for this finding. It is worth noting that CD4-positive T cells with CD10 expression in tissue samples do not necessarily indicate malignancy. CD10 expression on T cells, which has been reported in HIV and FL patients, was observed in reactive lymphoid hyperplasia and FL in our study. The expression of CD10 in AITL may have been overrated previously.

Using immunophenotypic abnormalities alone is not sufficient to differentiate the natures of T lymphocytes. Due to the polymorphistic phenotype of T lymphocytes, pan-T cell antigens may have changes in their expression intensity (inappropriately increased or decreased) under reactive conditions. T-cell clonality assessment is an important corollary examination for FCI to diagnose T-cell malignancies. However, the clonality of T cells cannot be identified easily, similar to B lymphocytes, using restricted expression of light chains. T-cell receptor (TCR) rearrangement is routinely used to identify the clonality of T cells. Nonneoplastic T-cell populations are polyclonal, whereas PTCLs are monoclonal. However, molecular analysis aims at all events instead of a single subgroup. TCR gene rearrangements are positive in 70–90% of AITL cases; moreover, nearly 10–20% of AITL cases are positive for Ig gene rearrangements (35). The Beta Mark TCR Vbeta Repertoire Kit and T-cell receptor (TCR) β-chain constant regions (TRBC1) enable the assessment of the clonality of TCR αβ T cells by flow cytometry. Compared with the TCR Vbeta Repertoire Kit, TRBC1 is a robust, cheap and labor- and specimen-saving antibody to identify the clonality of T cells (6, 36). In addition, the monoclonal expression of TRBC1 in CD8-positive T cells does not confirm that it is malignant (37). In the CD4-positive T-CUS, L-HES and TCR αβ negative AITL patients mentioned above, TRBC1 was negative. In consideration of the correlation between TRBC1, TCR αβ and CD3, we speculate that TRBC1 loss may be caused by loss of TCR αβ. But TRBC1 is still essential for screening AITL whether neoplastic T cells express TCR αβ or not, which is significant for distinguishing benign and malignant T cells. Polyclonal CD4-positive T cells with bright PD-1 expression can be frequently observed in tissue samples with RH, HL and FL. Under these non-T-cell malignancy circumstances, TRBC1 is of great importance for the exclusion of normal polyclonal T cells.

Flow cytometry would allow an efficient diagnostic workup, for it is good at determining the lineage, the stage, the cell of origin and the benign and malignant. However, in most cases, the judgment of the definite subtype is mainly relying on growth pattern and immunohistochemistry. For example, follicular T-cell lymphoma and nodal peripheral T-cell lymphoma with T follicular helper phenotype (TFH-PTCL) which didn't included in our paper. In fact, follicular T-cell lymphoma is a rare neoplasm and was rarely seen in our department. We had studied and discussed the diagnostic criteria of the 2017 WHO, and find that the points to distinguish AITL, follicular T-cell lymphoma and TFH-PTCL including CD4 expression, hyperplasia of FDCs or HEVs and EBV+ CD20+ B blasts are mainly determined by pathology. Since all of these three lymphomas have a TFH cell phenotype, we guess it may be very difficult for FC to differential diagnoses. Clinical history and other auxiliary examination may help. NGS studies have identified *IDH2 R172* mutations to be specific for AITL. About 20% of follicular T-cell lymphoma cases carry a t(5;9) (q33;q22) translocation, leading to *ITK-SYK* fusion, this translocation appears to be specific for follicular T-cell lymphoma (38, 39). Previous literatures speculate the TFH-PTCL may be a transitional phase of AITL or may constitute a tumor cell-rich variant of AITL because of their significant clinical and immunophenotypic overlap and plasticity, as well as similar TFH gene expression signature and mutation profiles (38, 40, 41). So in the 2022 WHO 5th, the above three TFH-derived T-NHLs are revised to: 1.Nodal TFH cell lymphoma, angioimmunoblastic-type; (2) Nodal TFH cell lymphoma, follicular-type; (3) Nodal TFH cell lymphoma, NOS (42). Till now there is no means can definitely distinguish or prove same of them, much more studies are required in future.

In this study, we demonstrated that using pan-T cell antibodies for screening AITL, as currently used in clinical practice, may underestimate the tumor burden. Accordingly, a rational procedure to differentiate AITL should begin with the exclusion of normal cell populations and the recognition of abnormal T cells in the context of normal hematopoietic cell populations. Bright expression of PD-1 was demonstrated on tumor cells in most cases other than diminished or absent CD3 and CD7 and it could clearly distinguish neoplastic T cells from normal/reactive CD4-positive T cells. In a few cases, detection improvement of neoplastic T-cell populations could be achieved by incorporating bright CD45 expression (and bright CD2 expression in tissue samples) and diminished expression of CD4 and CD5 (Figure 1). Nonetheless, AITL identification remains challenging by MFC in some cases, and more biomarkers should be explored.

In conclusion, T-cell screening tubes can reliably screen neoplastic T cells in AITL patients with typical immunophenotyping, such as loss of surface CD3 and loss of CD7, with a relatively high ratio. However, T-cell screening tubes are not sufficient to detect abnormal T cells when the expression of pan-T cell antigens is normal or they only have subtle changes. BM involvement has been underestimated. T-cell clonality identification is essential to exclude reactive T-cell populations. Using PD-1 and TRBC1 combined with pan-T cell antibodies can make a precise diagnosis of AITL and monitor minimal residual lesions regardless of the antigenic drift of neoplastic T cells.

## Data availability statement

The raw data supporting the conclusions of this article will be made available by the authors, without undue reservation.

## Ethics statement

The studies involving human participants were reviewed and approved by Ethics Committee, Tongji Hospital, Tongji Medical College, Huazhong University of Science and Technology, Wuhan, China. The patients/participants provided their written informed consent to participate in this study.

## Author contributions

CW wrote and submitted the manuscript. XM revised the manuscript. SL, SY, and LZ were responsible for data collection and flow cytometry. MX and YZ mainly discussed the manuscript. All authors contributed to the article and approved the submitted version.

## Conflict of interest

The authors declare that the research was conducted in the absence of any commercial or financial relationships that could be construed as a potential conflict of interest.

## Publisher's note

All claims expressed in this article are solely those of the authors and do not necessarily represent those of their affiliated organizations, or those of the publisher, the editors and the reviewers. Any product that may be evaluated in this article, or claim that may be made by its manufacturer, is not guaranteed or endorsed by the publisher.

## References

[1] Yabe M Dogan A Horwitz SM Moskowitz AJ. Angioimmunoblastic T-Cell Lymphoma. Cancer Treat Res. (2019) 176:99–126. 10.1007/978-3-319-99716-2_5 30596215

[2] Lunning MA Vose JM. Angioimmunoblastic T-cell lymphoma: the many-faced lymphoma. Blood. (2017) 129:1095–102. 10.1182/blood-2016-09-692541 28115369

[3] Swerdlow SH Campo E Harris NL Jaffe ES Pileri SA Stein H . (Eds): WHO *Classification of Tumours of Haematopoietic and Lymphoid Tissues (Revised 4th edition)*. IARC: Lyon (2017).

[4] Vose J Armitage J Weisenburger D. International peripheral T-cell and natural killer/T-cell lymphoma study: pathology findings and clinical outcomes. J Clin Oncol. (2008) 26:4124–30. 10.1200/jco.2008.16.4558 18626005

[5] Shi M Jevremovic D Otteson GE Timm MM Olteanu H Horna P. Single antibody detection of T-cell receptor αβ clonality by flow cytometry rapidly identifies mature T-cell neoplasms and monotypic small CD8-positive subsets of uncertain significance. Cytometry B Clin Cytom. (2020) 98:99–107. 10.1002/cyto.b.21782 30972977

[6] Berg H Otteson GE Corley H Shi M Horna P Jevremovic D . Flow cytometric evaluation of TRBC1 expression in tissue specimens and body fluids is a novel and specific method for assessment of T-cell clonality and diagnosis of T-cell neoplasms. Cytometry B Clin Cytom. (2021) 100:361–9. 10.1002/cyto.b.21881 32333725

[7] Baseggio L Berger F Morel D Delfau-Larue MH Goedert G Salles G . Identification of circulating CD10 positive T cells in angioimmunoblastic T-cell lymphoma. Leukemia. (2006) 20:296–303. 10.1038/sj.leu.2404013 16341050

[8] Lee P-S Lin C-N Chuang S-S. Immunophenotyping of angioimmunoblastic T-Cell lymphomas by multiparameter flow cytometry. Pathology - Research and Practice. (2003) 199:539–45. 10.1078/0344-0338-00459 14533938

[9] Chen W Kesler MV Karandikar NJ McKenna RW Kroft SH. Flow cytometric features of angioimmunoblastic T-cell lymphoma. Cytometry B Clin Cytom. (2006) 70:142–8. 10.1002/cyto.b.20107 16572417

[10] Stacchini A Demurtas A Aliberti S Francia di Celle P Godio L Palestro G . The usefulness of flow cytometric CD10 detection in the differential diagnosis of peripheral T-cell lymphomas. Am J Clin Pathol. (2007) 128:854–64. 10.1309/MC7QRGPTV0LRR98X 17951210

[11] Singh A Schabath R Ratei R Stroux A Klemke CD Nebe T . Peripheral blood sCD3(-) CD4(+) T cells: a useful diagnostic tool in angioimmunoblastic T cell lymphoma. Hematol Oncol. (2014) 32:16–21. 10.1002/hon.2080 23798351

[12] Loghavi S Wang SA Medeiros LJ Jorgensen JL Li X Xu-Monette ZY . Immunophenotypic and diagnostic characterization of angioimmunoblastic T-cell lymphoma by advanced flow cytometric technology. Leuk Lymphoma. (2016) 57:2804–12. 10.3109/10428194.2016.1170827 27105079 PMC5142610

[13] Cutrona G Ferrarini M. Expression of CD10 by human T cells that undergo apoptosis both in vitro and in vivo. Blood. (2001) 97:2528. 10.1182/blood.v97.8.2528 11307776

[14] Cook JR Craig FE Swerdlow SH. Benign CD10-positive T cells in reactive lymphoid proliferations and B-cell lymphomas. Mod Pathol. (2003) 16:879–85. 10.1097/01.MP.0000084630.64243.D1 13679451

[15] Amé-Thomas P Hoeller S Artchounin C Misiak J Braza MS Jean R . CD10 delineates a subset of human IL-4 producing follicular helper T cells involved in the survival of follicular lymphoma B cells. Blood. (2015) 125:2381–5. 10.1182/blood-2015-02-625152 25733581 PMC4401349

[16] Xerri L Chetaille B Serriari N Attias C Guillaume Y Arnoulet C . Programmed death 1 is a marker of angioimmunoblastic T-cell lymphoma and B-cell small lymphocytic lymphoma/chronic lymphocytic leukemia. Hum Pathol. (2008) 39:1050–8. 10.1016/j.humpath.2007.11.012 18479731

[17] Dorfman DM Brown JA Shahsafaei A Freeman GJ. Programmed death-1 (PD-1) is a marker of germinal center-associated T cells and angioimmunoblastic T-cell lymphoma. Am J Surg Pathol. (2006) 30:802–10. 10.1097/01.pas.0000209855.28282.ce 16819321 PMC3137919

[18] Krishnan C Warnke RA Arber DA Natkunam Y. PD-1 expression in T-cell lymphomas and reactive lymphoid entities: potential overlap in staining patterns between lymphoma and viral lymphadenitis. Am J Surg Pathol. (2010) 34:178–89. 10.1097/PAS.0b013e3181cc7e79 20087161 PMC2819320

[19] Roncador G Garcia Verdes-Montenegro JF Tedoldi S Paterson JC Klapper W Ballabio E . Expression of two markers of germinal center T cells (SAP and PD-1) in angioimmunoblastic T-cell lymphoma.Haematologica. (2007) 92:1059–66. 10.3324/haematol.10864 17640856

[20] Basha BM Bryant SC Rech KL Feldman AL Vrana JA Shi M . Application of a 5 marker panel to the routine diagnosis of peripheral T-cell lymphoma with T-follicular helper phenotype. Am J Surg Pathol. (2019) 43:1282–90. 10.1097/pas.0000000000001315 31283630

[21] Yabe M Gao Q Ozkaya N Huet S Lewis N Pichardo JD . Bright PD-1 expression by flow cytometry is a powerful tool for diagnosis and monitoring of angioimmunoblastic T-cell lymphoma.Blood Cancer J. (2020) 10:32. 10.1038/s41408-020-0301-x 32144240 PMC7060322

[22] Brüggemann M White H Gaulard P Garcia-Sanz R Gameiro P Oeschger S . Powerful strategy for polymerase chain reaction-based clonality assessment in T-cell malignancies report of the BIOMED-2 concerted action BHM4 CT98-3936. Leukemia. (2007) 21:215–21; 10.1038/sj.leu.2404481 17170730

[23] Langerak AW van Den Beemd R Wolvers-Tettero IL Boor PP van Lochem EG Hooijkaas H . Molecular and flow cytometric analysis of the Vbeta repertoire for clonality assessment in mature TCRalphabeta T-cell proliferations. Blood. (2001) 98:165–73. 10.1182/blood.v98.1.165 11418476

[24] Maciocia PM Wawrzyniecka PA Philip B Ricciardelli I Akarca AU Onuoha SC . Targeting the T cell receptor β-chain constant region for immunotherapy of T cell malignancies. Nature Med. (2017) 23:1416–23. 10.1038/nm.4444 29131157

[25] Chen M Wang A Liu S Wu X Gong M Zhen J . Analysis of the Expression of the TRBC1 in T lymphocyte tumors. Indian J Hematol Blood Transfus. (2021) 37:271–9. 10.1007/s12288-020-01357-x 33867734 PMC8012472

[26] Craig FE Foon KA. Flow cytometric immunophenotyping for hematologic neoplasms. Blood. (2008) 111:3941–67. 10.1182/blood-2007-11-120535 18198345

[27] Barrena S Almeida J Del Carmen García-Macias M López A Rasillo A Sayagués JM . Flow cytometry immunophenotyping of fine-needle aspiration specimens: utility in the diagnosis and classification of non-Hodgkin lymphomas. Histopathology. (2011) 58:906–18. 10.1111/j.1365-2559.2011.03804.x 21438908

[28] Zheng Y Wan X Gui X Chen Y Gao L Zhang H Wang Y. Value of multi-parameter flow cytometry immunophenotyping in T/NK-cell neoplasms in cytology specimens: a retrospective study in Chinese. Pathol Res Pract. (2020) 3:2921. 10.1016/j.prp.2020.152921 32499093

[29] Marti GE Rawstron AC Ghia P Hillmen P Houlston RS Kay N et al. Diagnostic criteria for monoclonal B-cell lymphocytosis. Br J Haematol. (2005) 130:325–32. 10.1111/j.1365-2141.2005.05550.x 16042682

[30] Wang C Mao X Liu S He C Wang Y Zhu L . The early diagnostic dilemma in angioimmunoblastic T cell lymphoma with excessive plasma cells. Prolif Case Rep Med. (2021) 2021:9951122. 10.1155/2021/9951122 34326878 PMC8302404

[31] Grogg KL Morice WG Macon WR. Spectrum of bone marrow findings in patients with angioimmunoblastic T-cell lymphoma. Br J Haematol. (2007) 137:416–22. 10.1111/j.1365-2141.2007.06577.x 17488486

[32] Vonderheid EC Bigler RD Kotecha A Boselli CM Lessin SR Bernengo MG . Variable CD7 expression on T cells in the leukemic phase of cutaneous T cell lymphoma (Sézary syndrome). J Invest Dermatol. (2001) 117:654–62. 10.1046/j.1523-1747.2001.01456.x 11564173

[33] Pulitzer MP Horna P Almeida J. Sézary syndrome and mycosis fungoides: an overview, including the role of immunophenotyping. Cytometry B Clin Cytom. (2021) 100:132–8. 10.1002/cyto.b.21888 32516521 PMC9306215

[34] Shomali W Gotlib J. World Health Organization-defined eosinophilic disorders: 2019 update on diagnosis, risk stratification, and management. Am J Hematol. (2019) 94:1149–67. 10.1002/ajh.25617 31423623

[35] Sokol K Kartan S Johnson WT Alpdogan O Nikbakht N Haverkos BM . Extreme peripheral blood plasmacytosis mimicking plasma cell leukemia as a presenting feature of angioimmunoblastic T-cell lymphoma (AITL). Front Oncol. (2019) 9:509. 10.3389/fonc.2019.00509 31263679 PMC6584846

[36] Horna P Otteson GE Shi M Jevremovic D Yuan J Olteanu H. Flow cytometric evaluation of surface and cytoplasmic TRBC1 expression in the differential diagnosis of immature T-cell proliferations. Am J Clin Pathol. (2022) 157:64–72. 10.1093/ajcp/aqab098 34302330

[37] Shi M Olteanu H Jevremovic D He R Viswanatha D Corley H . T-cell clones of uncertain significance are highly prevalent and show close resemblance to T-cell large granular lymphocytic leukemia implications for laboratory diagnostics. Mod Pathol. (2020) 33:2046–57. 10.1038/s41379-020-0568-2 32404954

[38] Huang Y Moreau A Dupuis J Streubel B Petit B Le Gouill S . Peripheral T-cell lymphomas with a follicular growth pattern are derived from follicular helper T cells (TFH) and may show overlapping features with angioimmunoblastic T-cell lymphomas. Am J Surg Pathol. (2009) 33:682–90. 10.1097/PAS.0b013e3181971591 19295409 PMC4838638

[39] Streubel B Vinatzer U Willheim M Raderer M Chott A. Novel t(5;9) (q33;q22) fuses ITK to SYK in unspecified peripheral T-cell lymphoma. Leukemia. (2006) 20:313–8. 10.1038/sj.leu.2404045 16341044

[40] Dobay MP Lemonnier F Missiaglia E Bastard C Vallois D Jais JP . Integrative clinicopathological and molecular analyses of angioimmunoblastic T-cell lymphoma and other nodal lymphomas of follicular helper T-cell origin. Haematologica. (2017) 102:e148–51. 10.3324/haematol.2016.158428 28082343 PMC5395128

[41] Attygalle AD Cabeçadas J Gaulard P Jaffe ES de Jong D Ko YH . Peripheral T-cell and NK-cell lymphomas and their mimics; taking a step forward - report on the lymphoma workshop of the XVIth meeting of the European association for haematopathology and the society for hematopathology. Histopathology. (2014) 64:171–99. 10.1111/his.12251 24128129 PMC6364972

[42] Alaggio R Amador C Anagnostopoulos I Attygalle AD Araujo IBO Berti E . The 5th edition of the world health organization classification of haematolymphoid tumours. Lymphoid Neoplasms Leukemia. (2022) 36:1720–48. 10.1038/s41375-022-01620-2 35732829 PMC9214472

