# Supplementary material for: PD-1 combined with TRBC1 and pan-T cell antibodies for robustly monitoring angioimmunoblastic T-cell lymphoma
Source: Front Med (Lausanne). 2022 Sep 8;9:962428. doi: 10.3389/fmed.2022.962428 (PMC9492947; doi:10.3389/fmed.2022.962428)
Supplement: Supplementary file 1 [file Data_Sheet_1.PDF]

HES 1

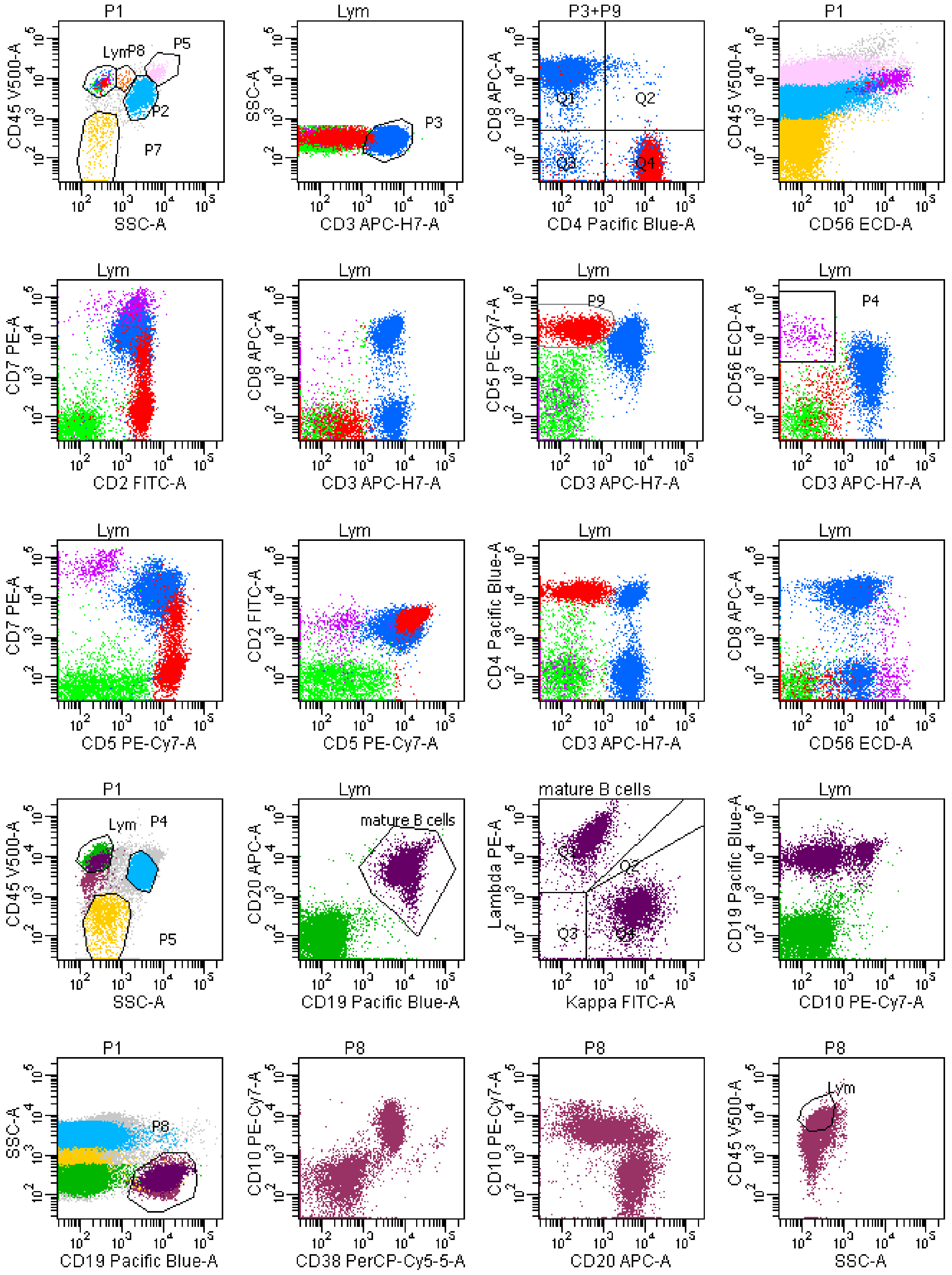
P1
CD45 V500-A
SSC-A
Lym
P8
P5
P2
P7
Lym
SSC-A
CD3 APC-H7-A
P3
P3+P9
CD8 APC-A
CD4 Pacific Blue-A
Q1
Q2
Q3
Q4
P1
CD45 V500-A
CD56 ECD-A
Lym
CD7 PE-A
CD2 FITC-A
Lym
CD8 APC-A
CD3 APC-H7-A
Lym
CD5 PE-Cy7-A
CD3 APC-H7-A
P9
Lym
CD56 ECD-A
CD3 APC-H7-A
P4
Lym
CD7 PE-A
CD5 PE-Cy7-A
Lym
CD2 FITC-A
CD5 PE-Cy7-A
Lym
CD4 Pacific Blue-A
CD3 APC-H7-A
Lym
CD8 APC-A
CD56 ECD-A
P1
CD45 V500-A
SSC-A
Lym
P4
P5
Lym
CD20 APC-A
CD19 Pacific Blue-A
mature B cells
mature B cells
Lambda PE-A
Kappa FITC-A
Q1
Q2
Q3
Q4
Lym
CD19 Pacific Blue-A
CD10 PE-Cy7-A
P1
SSC-A
CD19 Pacific Blue-A
P8
P8
CD10 PE-Cy7-A
CD38 PerCP-Cy5-5-A
P8
CD10 PE-Cy7-A
CD20 APC-A
P8
CD45 V500-A
SSC-A
Lym

HES 1

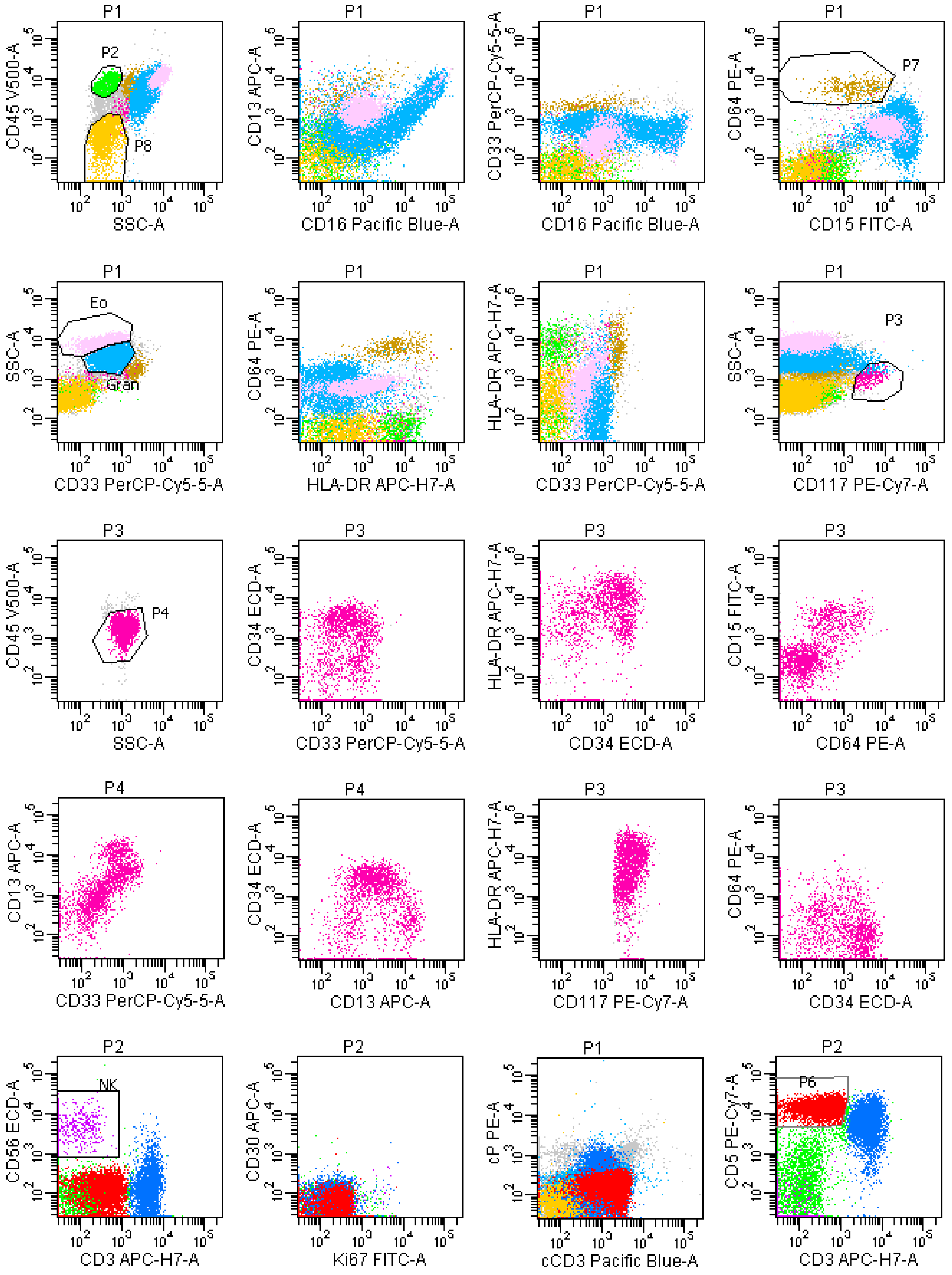
P1
P2
P8
CD45 V500-A
SSC-A
CD13 APC-A
CD16 Pacific Blue-A
CD33 PerCP-Cy5-5-A
P7
CD64 PE-A
CD15 FITC-A
Eo
Gran
HLA-DR APC-H7-A
P3
CD117 PE-Cy7-A
P4
CD34 ECD-A
CD56 ECD-A
NK
CD3 APC-H7-A
CD30 APC-A
Ki67 FITC-A
cP PE-A
cCD3 Pacific Blue-A
P6
CD5 PE-Cy7-A

HES 1

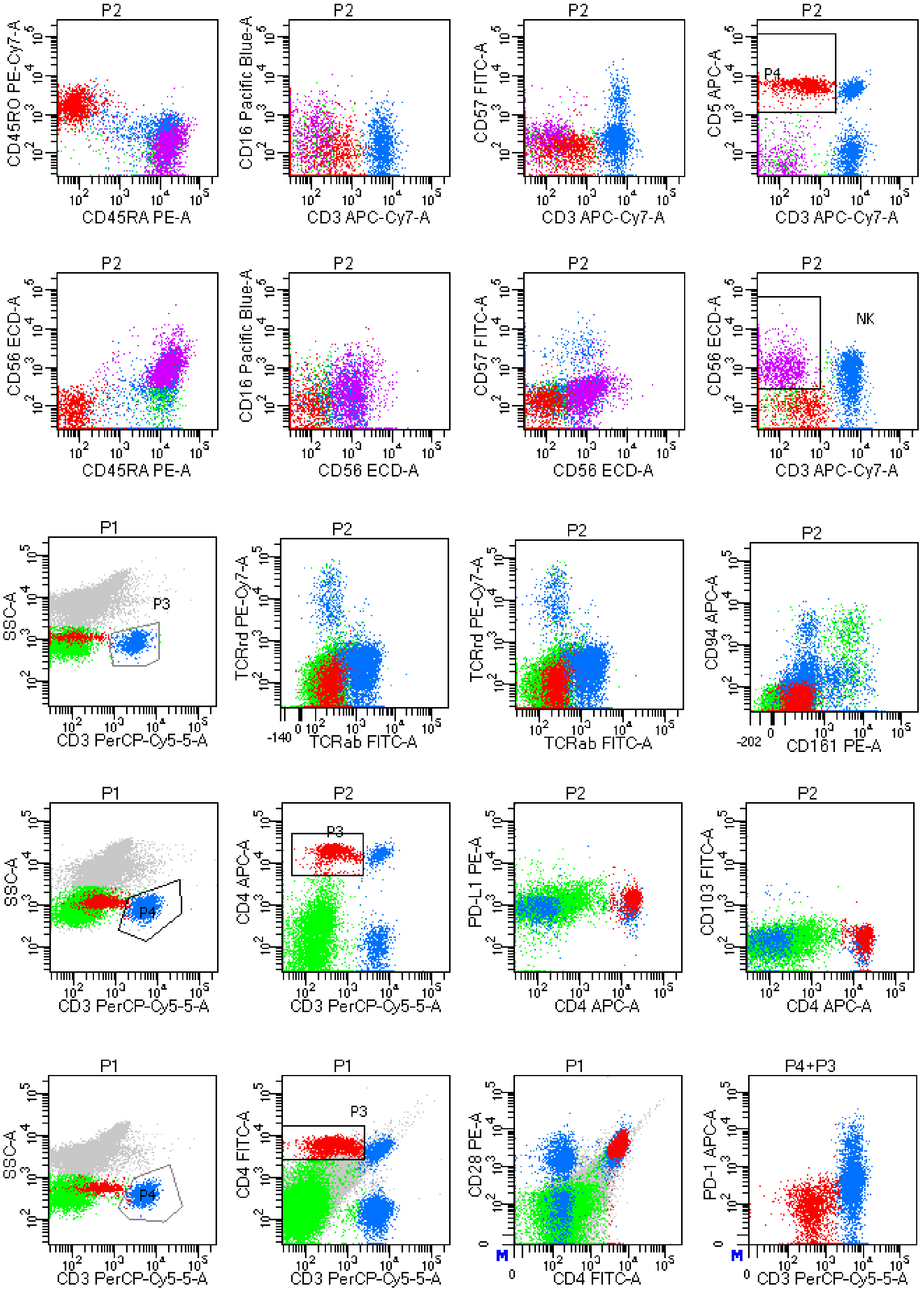
P2
CD45RO PE-Cy7-A
CD45RA PE-A
P2
CD16 Pacific Blue-A
CD3 APC-Cy7-A
P2
CD57 FITC-A
CD3 APC-Cy7-A
P2
P4
CD5 APC-A
CD3 APC-Cy7-A
P2
CD56 ECD-A
CD45RA PE-A
P2
CD16 Pacific Blue-A
CD56 ECD-A
P2
CD57 FITC-A
CD56 ECD-A
P2
NK
CD56 ECD-A
CD3 APC-Cy7-A
P1
P3
SSC-A
CD3 PerCP-Cy5-5-A
P2
TCRrd PE-Cy7-A
TCRab FITC-A
P2
TCRrd PE-Cy7-A
TCRab FITC-A
P2
CD94 APC-A
CD161 PE-A
P1
P4
SSC-A
CD3 PerCP-Cy5-5-A
P2
P3
CD4 APC-A
CD3 PerCP-Cy5-5-A
P2
PD-L1 PE-A
CD4 APC-A
P2
CD103 FITC-A
CD4 APC-A
P1
P4
SSC-A
CD3 PerCP-Cy5-5-A
P1
P3
CD4 FITC-A
CD3 PerCP-Cy5-5-A
P1
CD28 PE-A
CD4 FITC-A
P4+P3
PD-1 APC-A
CD3 PerCP-Cy5-5-A

HES 1

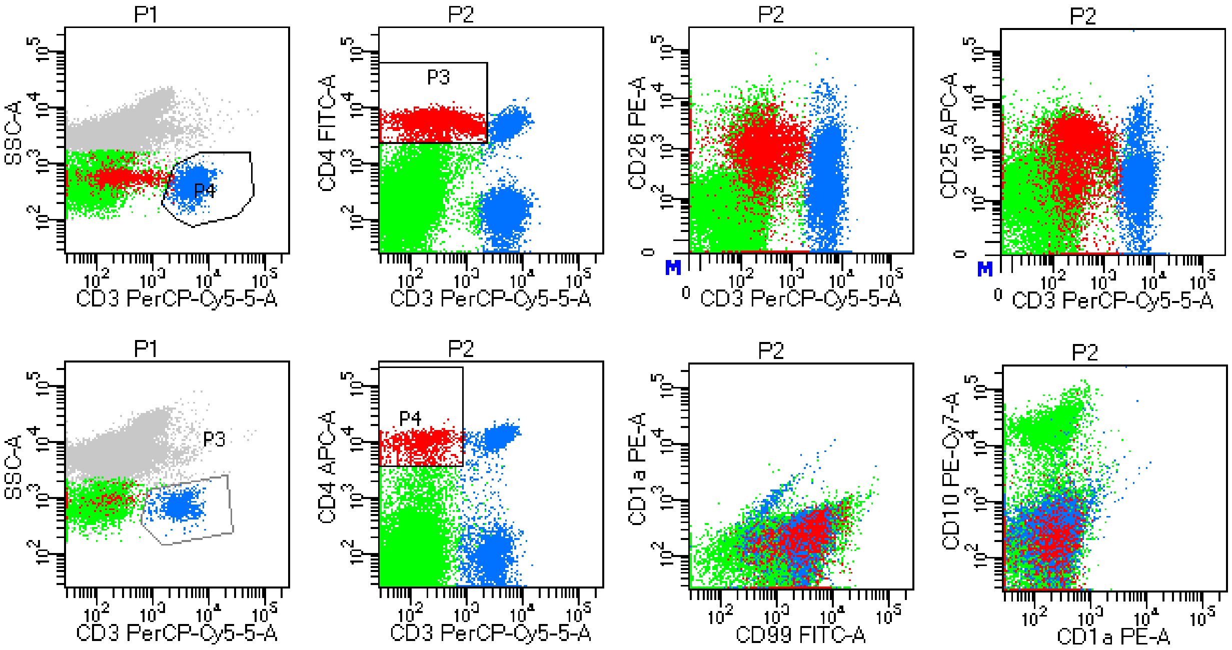
P1
SSC-A
CD3 PerCP-Cy5-5-A
P4
P2
CD4 FITC-A
P3
CD3 PerCP-Cy5-5-A
P2
CD26 PE-A
CD3 PerCP-Cy5-5-A
P2
CD25 APC-A
CD3 PerCP-Cy5-5-A
P1
SSC-A
P3
CD3 PerCP-Cy5-5-A
P2
CD4 APC-A
P4
CD3 PerCP-Cy5-5-A
P2
CD1a PE-A
CD99 FITC-A
P2
CD10 PE-Cy7-A
CD1a PE-A

HES 1

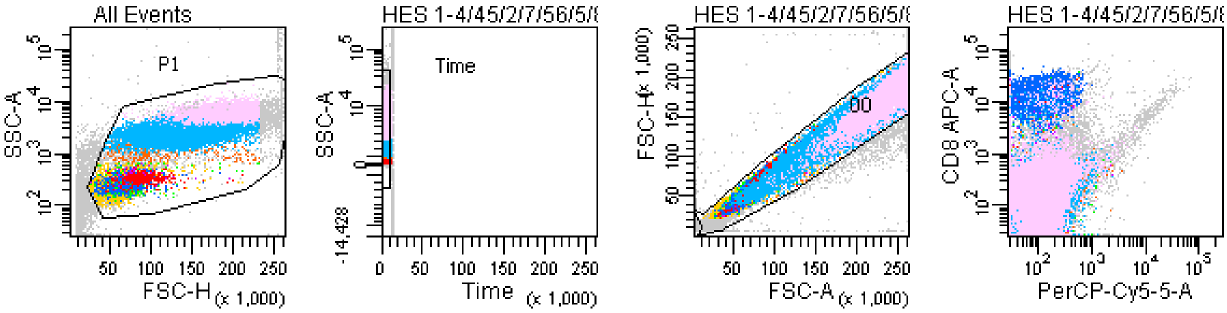

Tube: 4/45/2/7/56/5/8/3

Population	#Events	%Parent	%Total
All Events	521,533	####	100.0
Time	465,531	89.3	89.3
00	433,684	93.2	83.2
P1	394,237	90.9	75.6
Lym	29,778	7.6	5.7
P3	11,475	38.5	2.2
Q1	7,300	63.6	1.4
Q2	152	1.3	0.0
Q3	774	6.7	0.1
Q4	3,249	28.3	0.6
P4	1,002	3.4	0.2
P9	6,599	22.2	1.3
P7	35,925	9.1	6.9
[illegible]	[illegible]	[illegible]	[illegible]

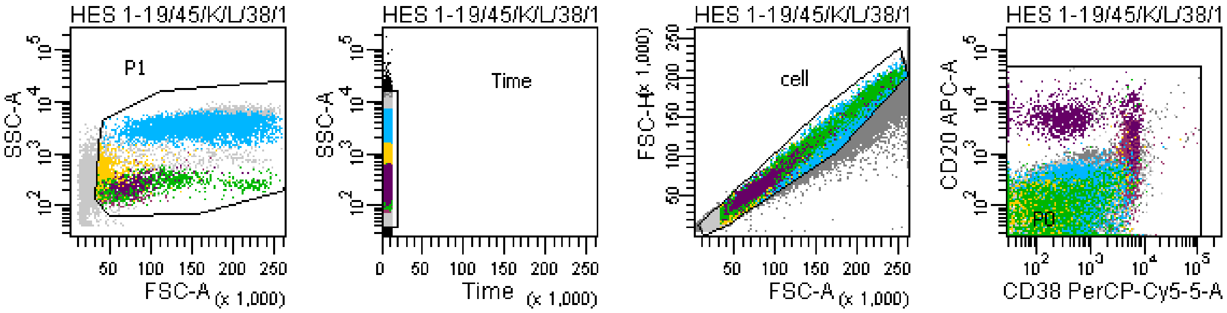

Tube: 19/45/K/L/38/10/20

Population	#Events	%Parent	%Total
All Events	300,000	####	100.0
Time	296,525	98.8	98.8
cell	200,101	67.5	66.7
P0	200,098	100.0	66.7
P1	166,714	83.3	55.6
Lym	24,221	14.5	8.1
mature B cells	5,455	22.5	1.8
Q1	2,369	43.4	0.8
Q2	6	0.1	0.0
Q3	171	3.1	0.1
Q4	2,909	53.3	1.0
P8	10,218	6.1	3.4
P4	94,924	56.9	31.6
P5	16,500	9.9	5.5

HES 1

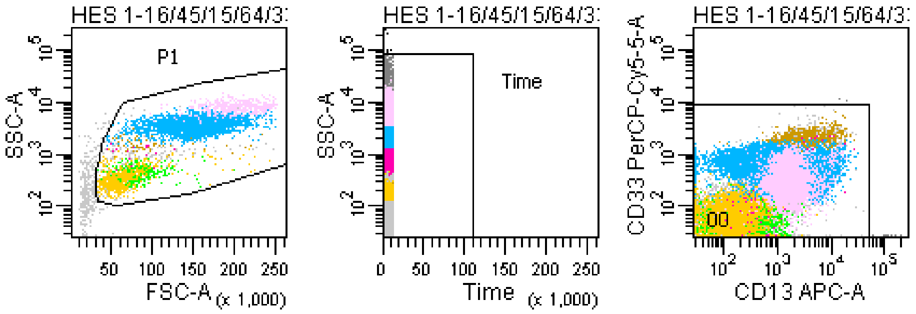

Tube: 16/45/15/64/33/34/117/13/DR

Population	#Events	%Parent	%Total
All Events	556,548	####	100.0
Time	556,541	100.0	100.0
cell	457,633	82.2	82.2
00	457,505	100.0	82.2
P1	417,777	91.3	75.1
P2	48,334	11.6	8.7
P8	50,693	12.1	9.1
P7	9,290	2.2	1.7
P3	2,237	0.5	0.4
P4	2,120	94.8	0.4
Gran	237,934	57.0	42.8
Eo	44,088	10.6	7.9

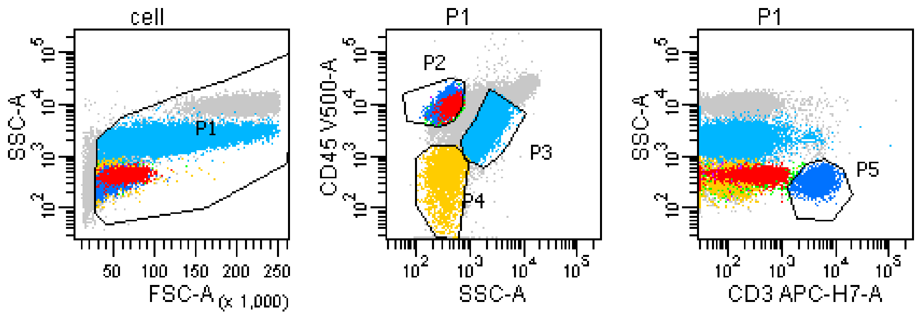

Tube: c3/45/Ki67/cP/56/5/30/3

Population	#Events	%Parent	%Total
All Events	568,739	####	100.0
Time	550,281	96.8	96.8
cell	503,150	91.4	88.5
P1	454,084	90.2	79.8
P2	39,190	8.6	6.9
P5	13,987	35.7	2.5
NK	1,508	3.8	0.3
P6	11,400	29.1	2.0
P3	321,386	70.8	56.5
P4	14,715	3.2	2.6

HES 1

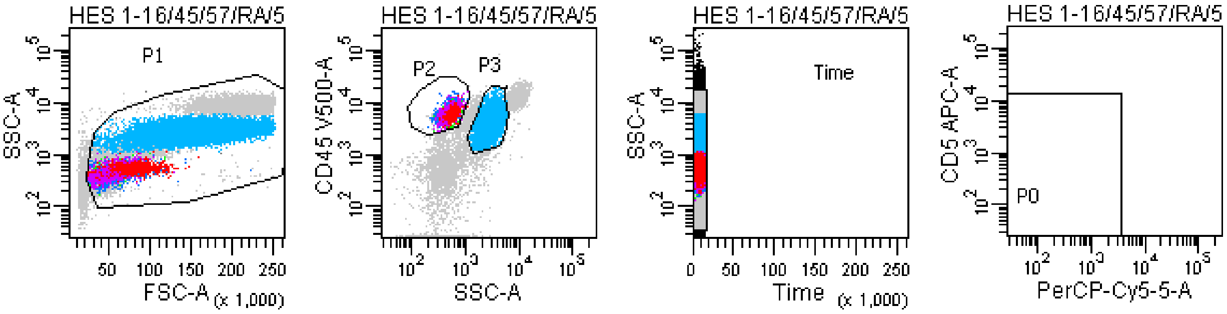

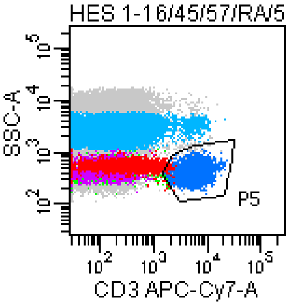

Tube: 16/45/57/RA/56/RO/4/3

Population	#Events	%Parent	%Total
All Events	471,456	####	100.0
Time	469,416	99.6	99.6
cell	418,636	89.2	88.8
P0	418,553	100.0	88.8
P1	400,402	95.7	84.9
P2	29,469	7.4	6.3
P5	10,214	34.7	2.2
NK	8,442	28.6	1.8
P4	7,233	24.5	1.5
P3	281,425	70.3	59.7

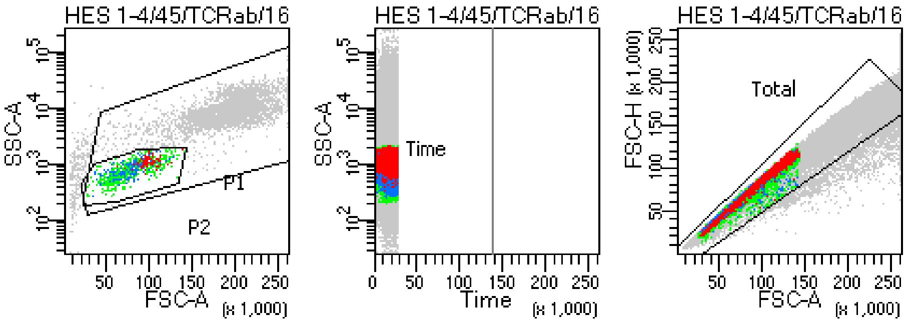

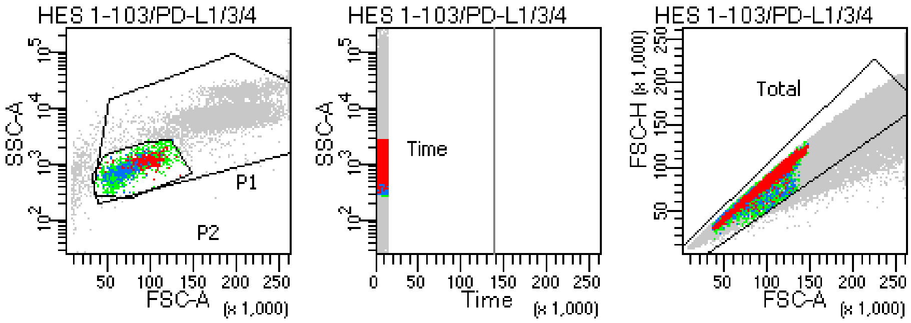

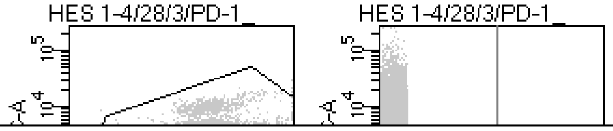

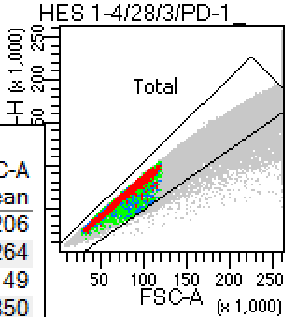

Population	#Events	%Parent	CD3 PerCP... Mean	PD-1 APC-A Mean
P1	593,307	94.5	548	1,206
P2	126,921	21.4	1,106	264
P3	13,207	10.4	558	49
P4	23,263	18.3	5,381	850

HES 1

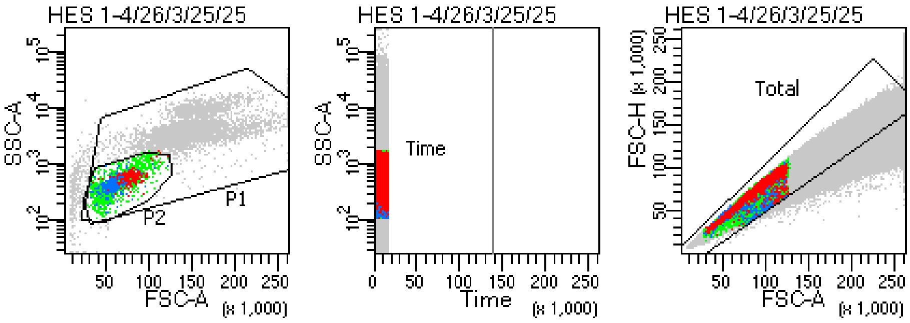

Tube: 4/26/3/25/25

Population	#Events	%Parent	%Total
All Events	1,000,000	####	100.0
Time	1,000,000	100.0	100.0
Total	962,587	96.3	96.3
P1	919,070	95.5	91.9
P2	207,113	22.5	20.7
P3	21,008	10.1	2.1
P4	33,263	16.1	3.3

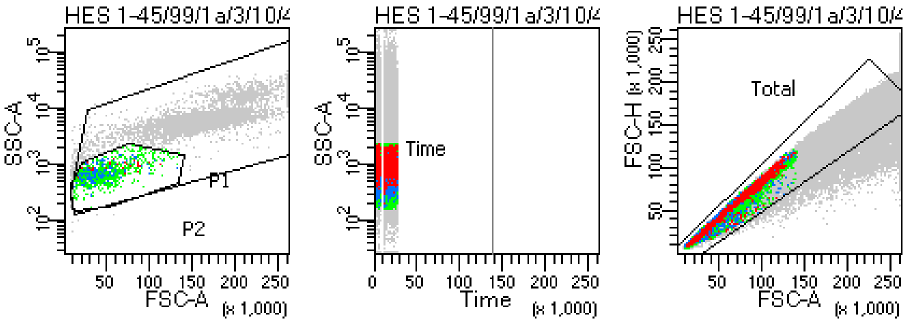

Tube: 45/99/1a/3/10/4

Population	#Events	%Parent	%Total
All Events	627,758	####	100.0
Time	627,758	100.0	100.0
Total	600,873	95.7	95.7
P1	586,094	97.5	93.4
P2	89,314	15.2	14.2
P4	3,535	4.0	0.6
P3	14,621	16.4	2.3

Printed on: 15, 2022 07:11:52 CST
