# Supplementary material for: PD-1 combined with TRBC1 and pan-T cell antibodies for robustly monitoring angioimmunoblastic T-cell lymphoma
Source: Front Med (Lausanne). 2022 Sep 8;9:962428. doi: 10.3389/fmed.2022.962428 (PMC9492947; doi:10.3389/fmed.2022.962428)
Supplement: Supplementary file 2 [file Data_Sheet_2.PDF]

HES 2

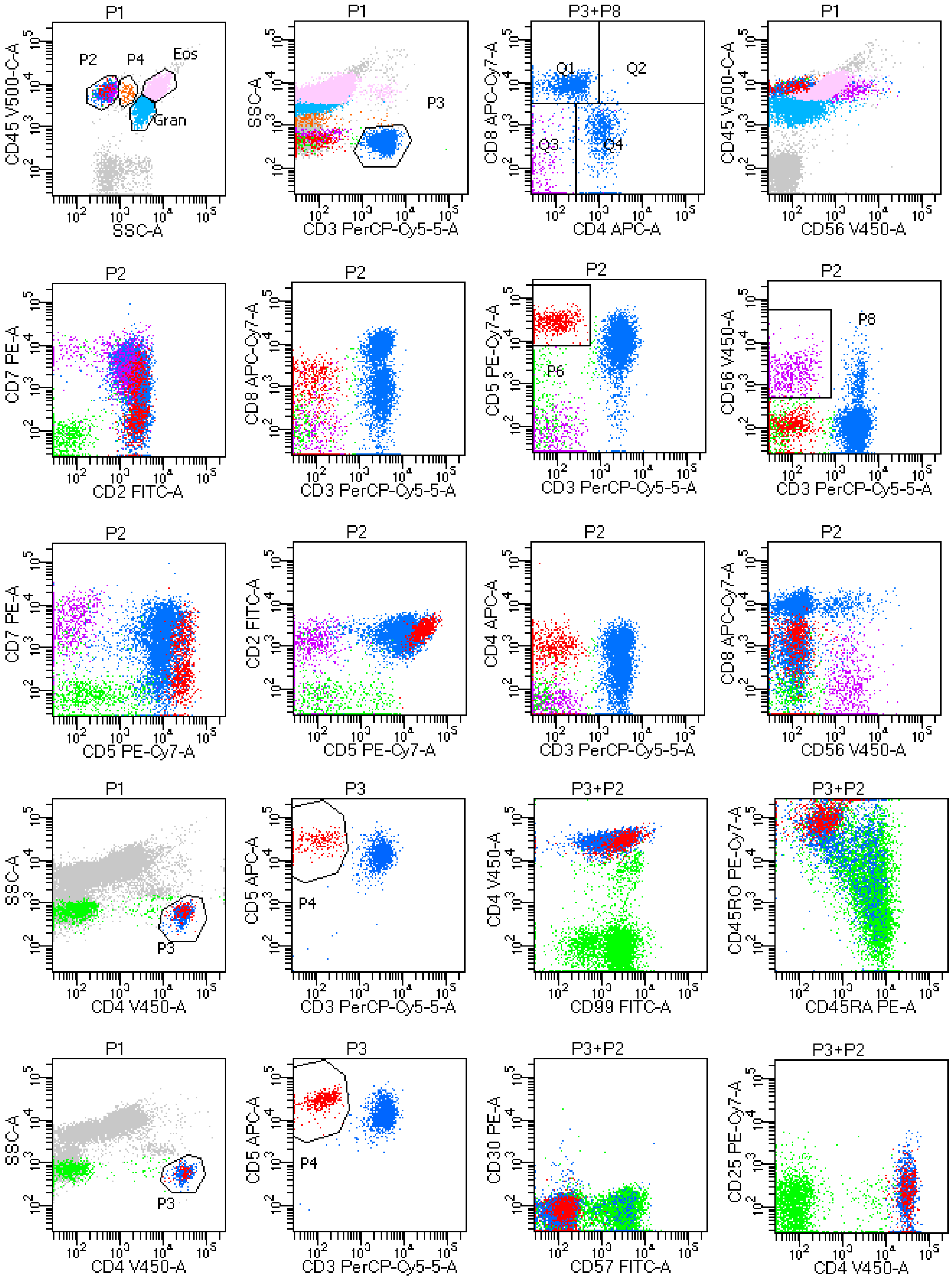

P1
P2
P4
Eos
Gran
CD45 V500-C-A
SSC-A
P1
SSC-A
P3
CD3 PerCP-Cy5-5-A
P3+P8
Q1
Q2
Q3
Q4
CD8 APC-Cy7-A
CD4 APC-A
P1
CD45 V500-C-A
CD56 V450-A
P2
CD7 PE-A
CD2 FITC-A
P2
CD8 APC-Cy7-A
CD3 PerCP-Cy5-5-A
P2
P6
CD5 PE-Cy7-A
CD3 PerCP-Cy5-5-A
P2
P8
CD56 V450-A
CD3 PerCP-Cy5-5-A
P2
CD7 PE-A
CD5 PE-Cy7-A
P2
CD2 FITC-A
CD5 PE-Cy7-A
P2
CD4 APC-A
CD3 PerCP-Cy5-5-A
P2
CD8 APC-Cy7-A
CD56 V450-A
P1
SSC-A
P3
CD4 V450-A
P3
CD5 APC-A
P4
CD3 PerCP-Cy5-5-A
P3+P2
CD4 V450-A
CD99 FITC-A
P3+P2
CD45RO PE-Cy7-A
CD45RA PE-A
P1
SSC-A
P3
CD4 V450-A
P3
CD5 APC-A
P4
CD3 PerCP-Cy5-5-A
P3+P2
CD30 PE-A
CD57 FITC-A
P3+P2
CD25 PE-Cy7-A
CD4 V450-A

HES 2

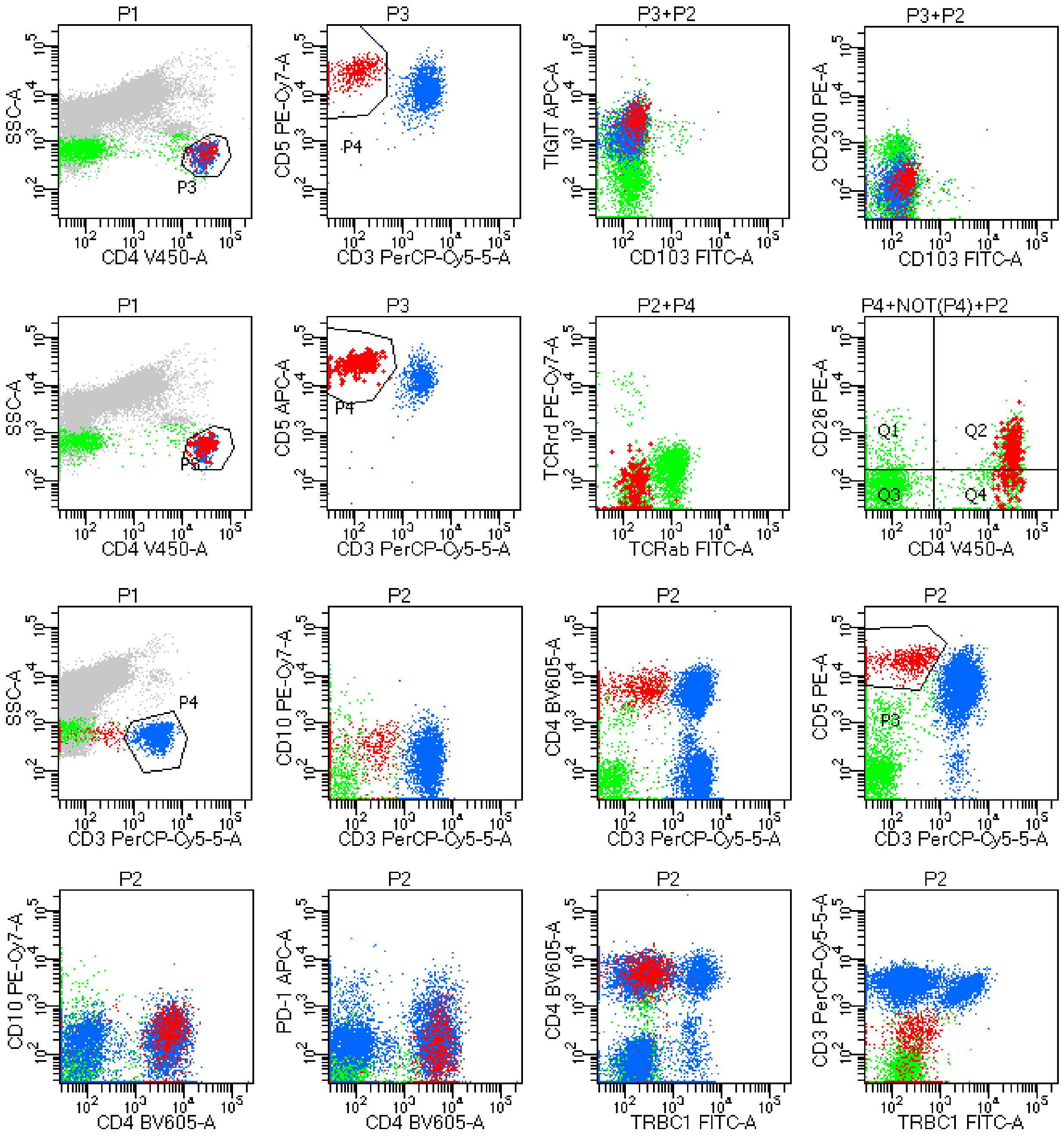
P1
SSC-A
CD4 V450-A
P3
CD5 PE-Cy7-A
CD3 PerCP-Cy5-5-A
P4
P3+P2
TIGIT APC-A
CD103 FITC-A
CD200 PE-A
CD5 APC-A
P2+P4
TCRrd PE-Cy7-A
TCRab FITC-A
P4+NOT(P4)+P2
CD26 PE-A
Q1
Q2
Q3
Q4
P2
CD10 PE-Cy7-A
CD4 BV605-A
CD5 PE-A
PD-1 APC-A
TRBC1 FITC-A
CD3 PerCP-Cy5-5-A

HES 2

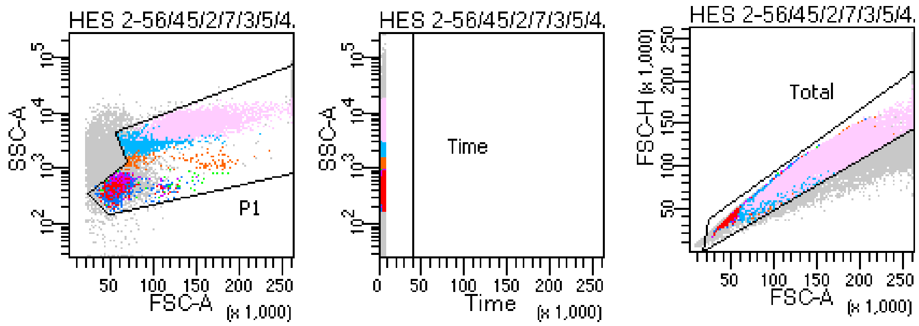

Tube: 56/45/2/7/3/5/4/8

Population	#Events	%Parent	%Total
All Events	369,720	####	100.0
Time	369,720	100.0	100.0
Total	346,831	93.8	93.8
P1	306,066	88.2	82.8
P2	14,711	4.8	4.0
P3	9,450	64.2	2.6
Q1	4,658	49.3	1.3
Q2	59	0.6	0.0
Q3	305	3.2	0.1
Q4	4,428	46.9	1.2
P8	1,783	12.1	0.5
P6	1,400	9.5	0.4
P4	1,779	0.6	0.5
Gran	47,798	15.6	12.9
Eos	208,683	68.2	56.4

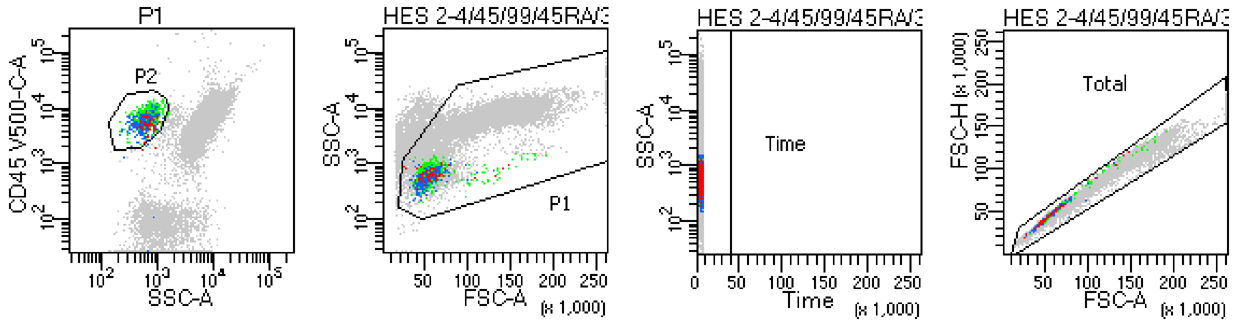

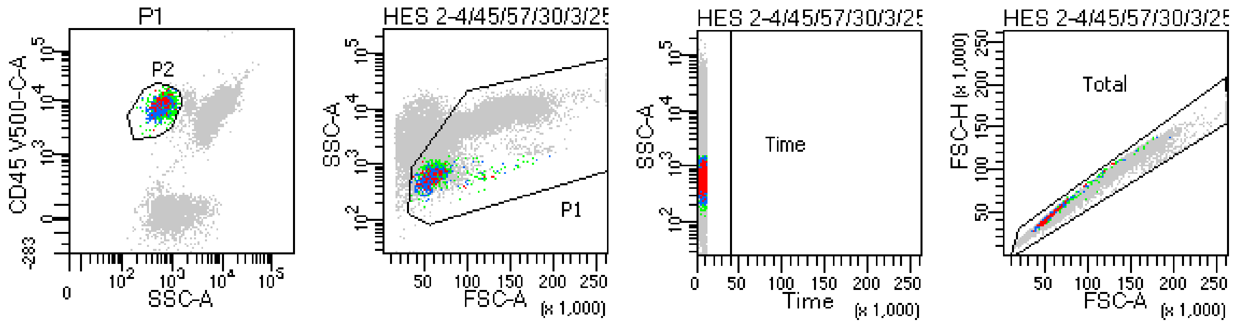

HES 2

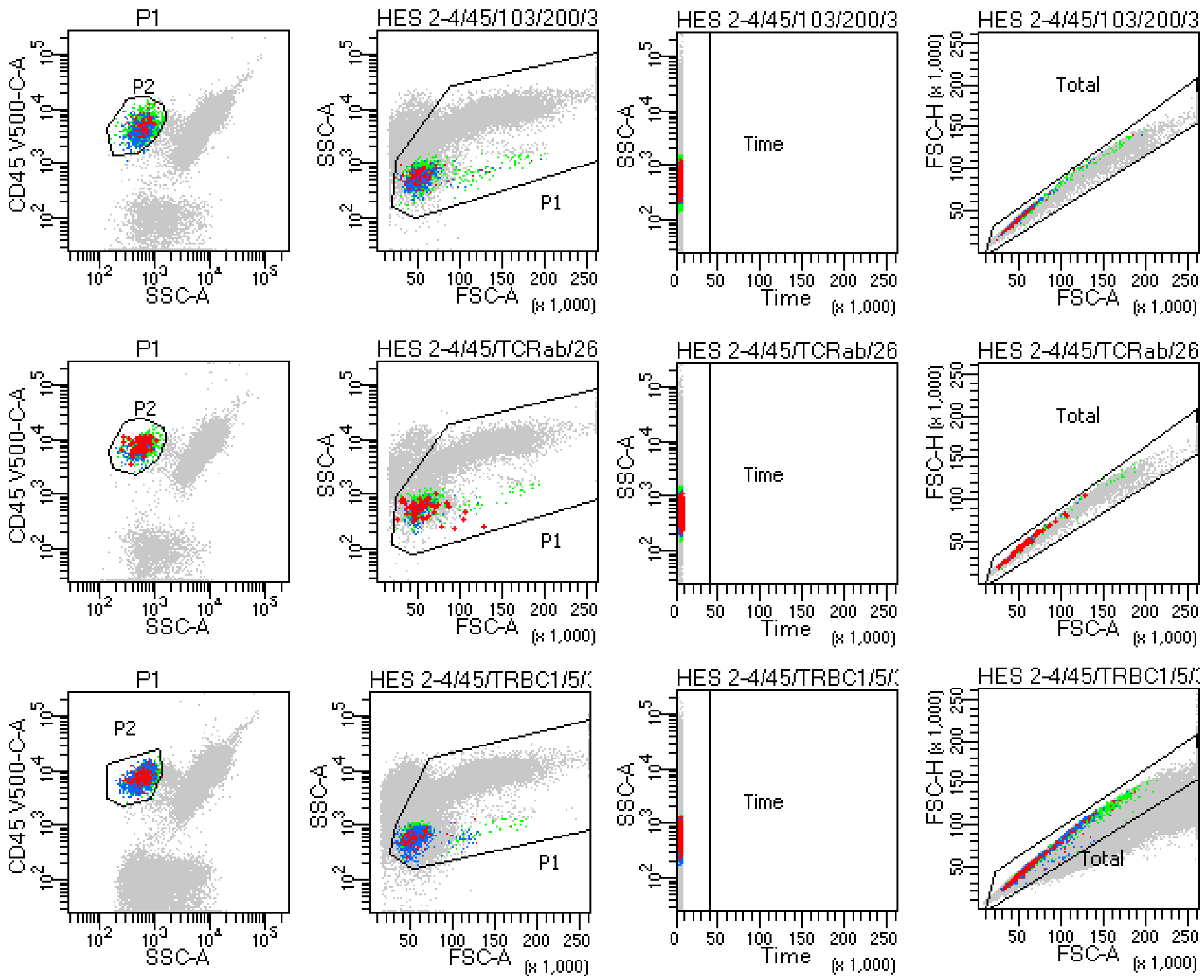

Tube: 4/45/TRBC1/5/3/10/PD-1

Population	#Events	%Parent	%Total
All Events	500,000	####	100.0
Time	500,000	100.0	100.0
Total	471,984	94.4	94.4
P1	430,138	91.1	86.0
P2	13,963	3.2	2.8
P3	946	6.8	0.2
P4	8,768	62.8	1.8
